# Geographic origin and evolutionary history of China’s two predominant HIV-1 circulating recombinant forms, CRF07_BC and CRF08_BC

**DOI:** 10.1038/srep19279

**Published:** 2016-01-14

**Authors:** Yi Feng, Yutaka Takebe, Huamian Wei, Xiang He, Jenny H. Hsi, Zhenpeng Li, Hui Xing, Yuhua Ruan, Yao Yang, Fan Li, Jing Wei, Xingguang Li, Yiming Shao

**Affiliations:** 1 State Key Laboratory for Infectious Disease Prevention and Control, National Center for AIDS/STD Control and Prevention, Chinese Center for Disease Control and Prevention, Collaborative Innovation Center for Diagnosis and Treatment of Infectious Diseases, Beijing; 2 AIDS Research Center, National Institute of Infectious Diseases, Tokyo, Japan; 3 Guangdong Provincial Institute of Public Health, Guangdong Provincial Center for Disease Control and Prevention, Guangzhou 511430, China

## Abstract

To determine the origin and evolutionary history of two predominant and closely-related circulating recombinant forms (CRF07_BC and CRF08_BC), recombinant structures and phylogenies of 7 unique recombinant forms comprised of subtypes of B’ (Thai B linage) and C (designated URFs_BC) from archival specimens of injection drug users (IDUs) collected in 1996 to 1998 from western Yunnan and 4 circulating recombinant forms with B’/C recombinants recently identified (designated nCRFs_BC) in China were compared with those of CRF07_BC and CRF08_BC. The results showed that 5 of 7 URFs_BC and all the nCRFs_BC shared recombination breakpoints with CRF07_BC and/or CRF08_BC. Yunnan URFs_BC consistently occupied the basal branch positions compared with CRF07_BC, CRF08_BC, and nCRFs_BC in phylogenetic trees. The estimated most recent common ancestors (tMRCA) for Yunnan URFs_BC were from ~1987, approximately half a decade earlier than those for CRF07_BC (~1994) and CRF08_BC (~1992). Discrete phylogeographic and spatial diffusion analysis revealed that both CRF07_BC and CRF08 BC came from western Yunnan in the early 1990s. Our results provide compelling evidence for western Yunnan as the geographic origin of CRF07_BC and CRF08_BC, which emerged from a swarm of URFs_BC by a series of recombination events in western Yunnan in the early 1990s.

After the first case of AIDS in mainland China was reported from an American tourist in 1985^1^,^2^, the HIV epidemic in China has become increasingly genetically complex, with many subtypes and recombinant forms of strains co-circulating in the past 30 years. In particular, four predominant strains, CRF07_BC, CRF08_BC, CFR01_AE, and B’ (Thailand variant of subtype B, also referred to as Thai B), were estimated to have resulted in more than 90% of the total infections in China^3^. Dehong prefecture in Yunnan Province, southwest China, which borders on Myanmar, close to the heroin-producing Golden Triangle, is considered one of the most important gateway of China’s HIV-1 epidemic, from where 3 (B’, CRF07_BC and CRF08_BC,) of the 4 most circulated HIV-1 strains in China were believed to have originated^4^,^5^,^6^,^7^,^8^,^9^,^10^,^11^,^12^. In 1989, HIV-1 subtype B’ was first found among 146 injection drug users (IDU) in Dehong prefecture^8^,^10^,^13^,^14^,^15^,^16^. Drug trafficking and abuse were considered to be the routes through which subtype B’ HIV-1 strains were brought in from Thailand and/or Myanmar^4^,^17^. Subsequently, the subtype B’ strain from this epidemic was found in contaminated plasma donations in central China resulting in an outbreak among plasma donors and their heterosexual contacts in Henan and neighboring provinces in mid-1990s^4^,^18^. Several years after subtype B’ was characterized in Dehong, subtype C HIV-1 strains presumably introduced from India were also identified in the same region^19^,^20^,^21^,^22^. For some unknown reasons, subtype C did not widely circulate, but the coexistence of subtypes B’ and C in Dehong IDUs provided an opportunity for the generation of various B’/C recombinant forms^9^,^23^, including two predominant and closely-related circulating recombinant forms (CRF07_BC^23^,^24^ and CRF08_BC^25^,^26^). CRF07_BC was initially detected among IDUs in Xinjiang, Gansu and Sichuan Province (bordering Yunnan in the North) of western China in 1996–1997^23^. Soon after that, CRF08_BC were found in IDUs sampled in 1997–1998 from Guangxi Province (bordering Yunnan in the East)^26^. In 2002, McCutchan *et al.*^7^ first reported that CRF07_BC and CRF08_BC shared several precise B’/C boundaries, suggesting that they shared a common ancestor, and Yunnan Province is likely the origin for both CRFs. However, in contrast with CRF08_BC, samples of CRF07_BC have rarely been collected from western Yunnan, and for years, no available CRF07_BC sequences from Yunnan could be found at the root place of the CRF07_BC cluster in the phylogenetic trees. A more recent study by Liu *et al.*^12^ argued the possibility that CRF07_BC originated from northwestern China, suggesting it may originated from Pakistan. None of the existing studies included enough early samples of the HIV-1 epidemic in Yunnan to provide a conclusive answer to the questions: Did both CRF07_BC and CRF08_BC originate from western Yunnan? How and when were CRF07_BC and CRF08_BC generated? What factors influenced the geographical origin and evolutionary history?

To investigate the early history of HIV-1 epidemic in western Yunnan, the near full-length genome (NFLG) sequences of the B’/C recombinants were determined from archival specimens sampled in 1996–1998 from western Yunnan. The evolutionary history of B’/C recombinants in China was reconstructed, and more compelling evidences were presented to support the idea that western Yunnan is most likely the birthplace of CRF07_BC and CRF08_BC.

## Results

### Early Yunnan B’/C recombinants screening

A total of 100 archival HIV-1 positive plasma specimens collected in 1996 and 1998 from consenting IDUs in western Yunnan were used for B’/C recombinant samples screening. The genotyping results for the sequences of the *env* C2V3 region showed that there were 90 subtype B’ and 10 subtype C samples. The near full-length genome (NFLG) sequences (HXB2: 552–9636 nt) were amplified from 20 samples including all 10 subtype C samples and 10 randomly-selected subtype B’ samples. Among them, 8 subtype C and 4 subtype B’ samples were successfully sequenced. All 4 NFLG sequences obtained from samples which were subtype B’ in the *env* region belong to non-recombinant forms of subtype B’ (pure subtype B’). One of the eight samples which was subtype C in the *env* region belongs to non-recombinant forms of subtype C (pure subtype C). The other 7 NFLG sequences obtained from subtype C samples were various forms of B’/C recombinants. The results were subsequently confirmed by the neighbor-joining tree analysis with MEGA 6.0 (Supplemental Figure S1). The mosaic structures of the 7 B’/C recombinants were different from each other and also were not identical to those of CRF07_BC, CRF08_BC and other B’/C recombinant strains previously identified (The genome region from 552 nt to 2642 nt of the 96YN3018 was not successfully sequenced).

### Recombinant structures of Yunnan B’/C recombinants and their relationship with CRF07_BC and CRF08_BC

**Figure 1:**
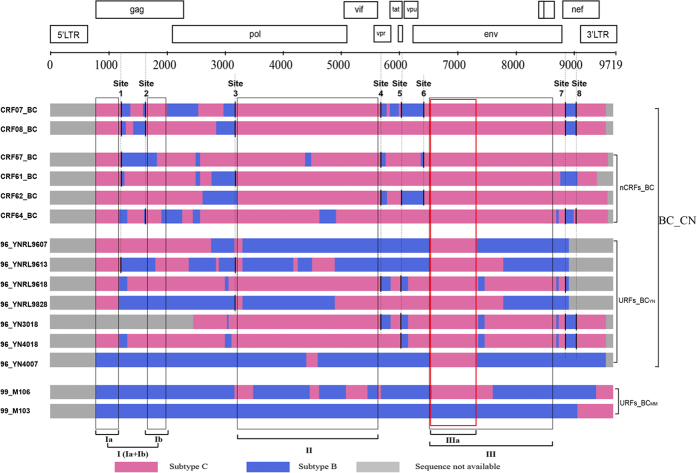
Precise mapping of recombination breakpoints in the B’/C recombinant forms in western Yunnan. Detailed subtype structure of the B’/C recombinant forms was estimated as described in Materials and Methods (see also Supplemental Figure S2, S3). Subtype B’ region (blue), subtype C region (magenta). The sites 1 through 8 are the recombination breakpoints shared among CRF07_BC, CRF08_BC, and other B’/C recombinants that are indicated with thick vertical lines. Regions Ia (HXB2: 790–1191), Ib (HXB2: 1642–2010 nt) II (HXB2: 3330–5593 nt). IIIa (HXB2: 6541–7348 nt), and III (HXB2: 6541–8695) are the subtype C segments that were selected for the subregion MCC tree analyses (see Fig. 2) and temporal dynamic analyses of spatial diffusion for B’/C recombinants in order to examine the relationships and the timescale of emergence and diffusion of CRF07_BC/CRF08_BC.

The breakpoints of all CRFs_BC characterized in China and URFs_BC sampled earlier than year 2000 from China and Myanmar were precisely defined by combining the results of Bootscan and Similarity plot (Supplemental Figure S2). The deduced subtype structures of the B’/C recombinant strains were carefully compared. The patterns of the shared recombination breakpoints are summarized in Fig. 1 and Table 1. To confirm the breakpoints we characterized, effective informative sites between subtypes around each breakpoint were carefully checked and the sites in the middle of two effective sites that refer to the reference sequences of subtype B’ and C were finally defined as recombination breakpoints (Supplemental Figure S3). Of note, some breakpoints of the CRFs_BC we characterized were not identical with the structures posted on the Los Alamos HIV sequence database. The updated subtype structures of the CRFs_BC are shown in Supplemental Figure S4. As shown in Fig. 1, there are 16 breakpoints in CRF07_BC and 8 in CRF08_BC. Five of these breakpoints are apparently shared between the two CRFs. All the breakpoints are identical with the distribution of McCutchan *et al.*^7^, There are several more (3 in CRF07_BC and 1 in CRF08_BC) short subtype B’ or C fragments of recombination (50 nt–200 nt) than the structures which are shown on the Los Alamos HIV sequence database. A number of recombination breakpoints in different genome regions (*gag*: 1215 and 1641, *pol*: 3180, *vpr*: 5694, *tat*: 6045, *env*: 6431 and *nef*: 8873 and 9059 based on the scale of HXB2) were shared among these CRFs_BC and URFs_BC (Table 1). For simplicity, the 8 shared recombination breakpoints were designated as Site 1 through Site 8. Sites 1, 2, 3, 7 and 8 were common breakpoints shared in both CRF07_BC and CRF08_BC. All four nCRFs_BC (CRF57_BC, CRF61_BC, CRF62_BC, and CRF64_BC) and five of the seven URFs_BC sampled in 1996–1998 from Yunnan (96_YN3018, 96_YNRL4018, 96_YNRL9613, 96_YNRL9618, and 98_YNRL9828,) share 1 to 4 breakpoints with CRF07_BC and/or CRF08_BC. None of these breakpoints were shared with the 2 Myanmar URFs. (Fig. 1, Table 1). The common sites Site 1 to 8 were shared 3 to 6 times among the CRFs_BC and URFs_BC (Fig. 1). These observations indicated that there should have been a “parental” B’/C recombinant which carried these breakpoints as the common ancestor of these B’/C recombinant lineages.

### Phylogenetic and evolutionary relationships of URFs_BC and nCRFs_BC with CRF07_BC and CRF08_BC

To explore the phylogenetic and evolutionary relationships of subtype C fragments in the B’/C recombinants, Bayesian phylogenetics were performed with molecular clock analyses using the relaxed molecular clock model. Since it was difficult to locate a longer region (>300 bps) of subtype B’ in either CRF07_BC or CRF08_BC in order to yield results with adequate statistical significance (see Fig. 1), we only used subtype C regions for analyses. A set of the subtype C regions were chosen in a way so as to maximize phylogenetic resolution while including the maximum possible numbers of sequences for analysis. The subtype C regions selected for the analyses were: Region I (concatenated Ia and Ib, Ia + Ib) [HXB2: 790–1191 nt + 1642–2010 nt (771-bp)] in the HIV-1 *gag* gene, Region II [HXB2: 3331–5593 nt (2,263-bp)] in the *pol* gene, Region IIIa [HXB2: 6541–7348 nt (808-bp)], and Region III [HXB2: 6541-7-8695 nt (2,154-bp)] in the *env* gene (Fig. 1). Each subtype C region was thus designed to test the phylogenetic and evolutionary relationships after excluding the segments containing subtype B’ in the regions (Region I (Ia + Ib), Region II, and Region III).

**Figure 2:**
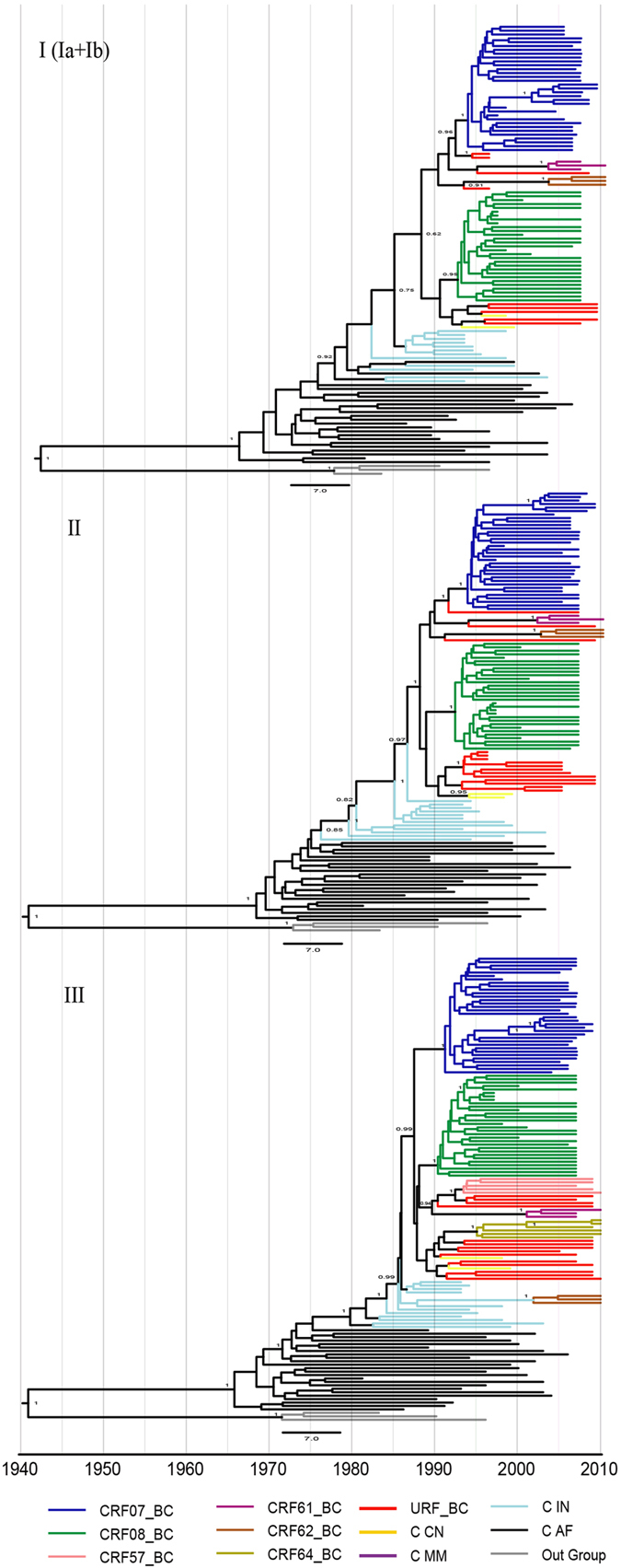
MCC trees based on selected subtype C segments considering B’/C recombinant forms in this study. Bayesian molecular clock analyses were performed using BEAST v1.74 (see methods for details) for different subtype C regions: (**A**) the concatenated *gag* region (Ia + Ib) (HXB2: 790–1191 nt and 1642–2010 nt), (**B**) the *pol* region (II) (3330–5593 nt), and (**C**) the *env* region (III) (6541–8695 nt).The tree branches are color-coded according to their respective genotypes and geographical locations. The medians of tMRCA are as follows: CRF07_BC ancestor = ~1994, CRF08_BC ancestor = ~1992, CRF57_BC ancestor = ~1993, CRF61_BC ancestor = ~2002, CRF62_BC ancestor = ~2003, CRF64_BC ancestor = ~1995, Yunnan RF ancestor = ~1987. Indian subtype C ancestor = ~1976, and African subtype C ancestor = ~1966 (see Table S1).

As shown in Fig. 2, maximum clade credibility (MCC) trees revealed that most of Yunnan URFs_BC were consistently placed at the basal position of the CRFs and were located under the branches of Indian subtype C in all the 3 subtype C regions. The subtype C segments of Yunnan URFs_BC thus exhibited intermediate characteristics connecting Indian subtype C and CRFs_BC in China.

**Figure 3:**
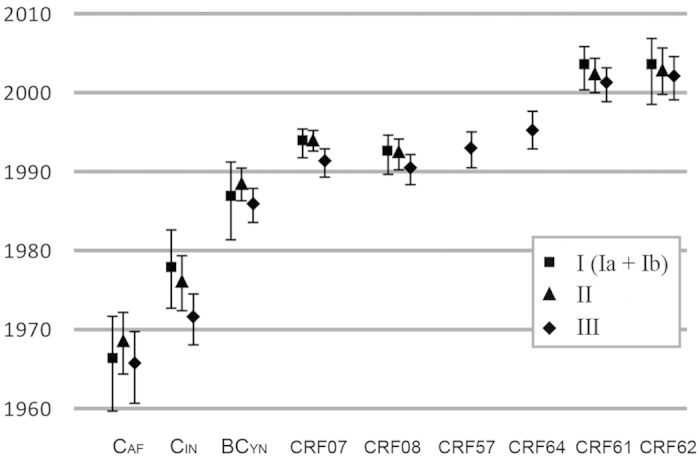
Estimated tMRCAs of subtype C and its recombinant lineages in China. Estimated dates of the MRCA of CRF07_BC and CRF08_BC in comparison with nCRFs_BC and ancestors of URFs_BC were obtained for sub-genomic regions. Estimates were calculated under a GTR substitution model with a relaxed molecular clock and a Bayesian skyline plot coalescent model. Error bars represent the 95% higher probability density credible regions for each estimate. Estimates obtained using region (Ia + Ib:), (II), and (III) are labeled with square, triangle, and diamond respectively.

The estimated evolutionary rates for Region I (Ia + Ib), II, and III were 3.76 (2.87–4.62) ×10^3^, 2.23 (1.96–2.49) ×10^3^ and 6.36 (5.24–7.96) ×10^3^substitutions/site per year respectively (numbers in parenthesis show the 95% highest posterior density for each estimate). The estimated tMRCA for subtype C and its B’/C recombinant lineages of Yunnan was ~1987, approximately more than half a decade older than those estimated for CRF07_BC (~1994) and CRF08_BC (~1992). CRF57_BC (~1993) and CRF64 (~1995) seem to have originated in the same time period as CRF07_BC and CRF08_BC, while CRF61_BC (~2002) and CRF62 (~2003) were estimated to have originated around 10 years later (Fig. 3, summarized in Table S1). The topology of MCC trees for each key node was highly consistent in all 3 regions except CRF62_BC in the tree of region III. As shown in Fig. 2 III, CRF62_BC was located in the branches of Indian C. This is likely due to additional recombination events between Yunnan URFs_BC and Indian C (Fig. 2).

**Figure 4:**
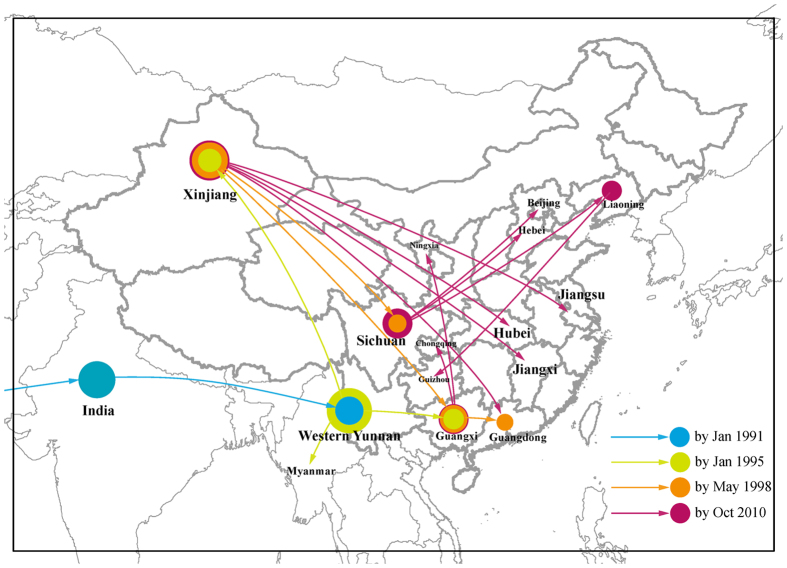
Temporal dynamics of spatial diffusion for B’/C recombinants in China and related “parental” subtype C lineages. The temporal dynamics of spatial diffusion for B’/C recombinants and related lineages can be displayed follow the software instructions of SPREAD^32^. The sketch map was drawn using Adobe Illustrator CS6 refer to the Bayesian inference of diffusion process estimated by SPREAD. The dispersal patterns for 1991, 1995, 1998, and 2010 are shown as different colors. Lines between locations represent branches in the MCC tree along which the relevant location transition occurs. Location circle diameters are proportional to the square root of the number of MCC branches maintaining a particular location state at each time-point.

In order to maximise the temporal evolutionary signal, Region IIIa (HXB2: 6541–7348) was selected as the optimal region for examining the discrete phylogeographic and spatial diffusion analysis since all the CRFs_BC and the seven URFs_BC sampled in 1996–1998 with one of Myanmar URFs_BC (99_M106) are subtype C in this region (Fig. 1). It contains most B’/C recombinants of our interest in the early stage when CRF07_BC and CRF08_BC were generated. This phylogeography implementation provided the migration process in natural time scales. Figure 4 was drawn based on the visualization of KML files (see Methods and Supplemental data) summarized the temporal dynamics of spatial diffusion for B’/C recombinants in China and their related “parental” subtype C lineages from India and Africa. The lines that connect different locations represent occurrence of exchanges and circular areas reflect the number of branches in the MCC tree at that time point. Subtype C strains of India originated from Africa in the mid-1960s, and were introduced to China in the mid-1980s. Subsequently, by 1991, subtype C and its B’/C recombination lineages had accumulated in western Yunnan, where subtype C segments were originally detected from IDUs in Longchuan and Ruili counties of Yunnan Province during 1992–1993^19^. Subtype B’ strains were also introduced into this population around the same time period^4^. It appears that B’/C recombinants were continuously generated by a series of recombination events in a setting of intermixing of subtype B’, C, and their recombination lineages. By 1995, CRF07_BC and CRF08_BC appear to have stood out from these recombination strains and spread to Xinjiang and Guangxi Provinces, respectively. The diffusion process intensified from 1998 and the virus reached more provinces such as Sichuan, Ningxia, Liaoning, Guangdong, and Beijing. Broadly circulating B’/C recombination strains resulted in severe nationwide outbreaks in China. Considering that CRF07_BC and CRF08_BC have infected hundreds of thousands people nationwide, the actual epidemic history of B’/C recombinants should be much more complicated than what we have shown in the sketch, but we can still reach the conclusion that Xinjiang, Guangxi, and Sichuan have been crucial in the process of the spread of the viruses in the early stage of the epidemic.

## Discussion

### Reconstruction of the early history of B and C recombinants HIV-1 in China by combining the evidences of phylogenetic and evolutionary relationships

The precision-optimized breakpoint analysis approaches and Bayesian phylogenetic molecular clock analyses were used in this study to provide new evidences that CRF07_BC and CRF08_BC had originated from B’/C recombinants generated among IDUs in western Yunnan, China. Many early HIV-1 B’/C recombinants circulated in Dehong IDUs shared breakpoints identical to those of CRF07_BC and CRF08_BC (Fig. 1, Table 1). Since recombination breakpoints are conserved during viral evolution, the shared breakpoints strongly suggest a direct evolutionary relationship. It is possible that these CRFs_BC and URFs_BC were generated from CRF07_BC and/or CRF08_BC by secondary recombination events, but the phylodynamics analyses of our study have ruled out this possibility. To locate longer regions with no recombinant in either CRF07_BC or CRF08_BC in order to maximize phylogenetic resolution, only subtype C regions from *gag*, *pol,* and *env* genes were selected for phylogenetic analyses. The estimated tMRCAs of Indian C for each subtype C region were calculated and dated to be around 1975, which is highly consistent with that of previous studies analyzing with different gene regions^5^,^21^,^22^. The results support the conclusion that the Indian subtype C originated from a single or a few South African lineages in the middle of 1970s^21^,^22^,^26^,^27^. Furthermore, the MCC trees in our analyses (Fig. 2) demonstrated that sequences of subtype C regions in the Yunnan URFs_BC consistently occupied the basal positions of CRF07_BC and CRF08_BC branches. The Bayesian molecular clock analyses and temporal dynamics of spatial diffusion for B’/C recombinants clearly show the timeline for the emergence and diffusion of subtype C and its recombinant lineages in China. This evidence corroborates the notion that CRF07_BC and CRF08_BC arose from B’/C recombinants generated in western Yunnan^5^,^6^,^23^.

Various historical epidemiological data support our conclusion^5^,^6^,^9^,^11^. The Chinese national surveillance in 1989 detected the first Chinese HIV outbreak among IDUs in Dehong^8^,^10^,^13^. The estimated tMRCA of Yunnan subtype C and its B’/C recombinants (~1987) is approximately 2 years prior to the discovery of the outbreak in the national surveillance. In several precedent studies, the tMRCA for subtype B’ in China was estimated in ~1985, very close to the estimated tMRCA for Yunnan subtype C and its B’/C recombinants^4^,^18^,^28^. It suggests that recombination between subtypes B’ and C in western Yunnan likely began soon after the epidemic among IDUs started in this region.

However, in contrast to CRF08_BC, samples of CRF07_BC have rarely been collected from western Yunnan, and for years no available CRF07_BC sequences from Yunnan could be found at the root place of the CRF07_BC cluster in the phylogenetic trees. Furthermore, existing studies had not obtained enough early samples of the HIV-1 epidemic in Yunnan to provide a conclusive answer for the question of whether CRF07_BC originated from western Yunnan or not. Liu *et al.*^12^ recently found, through analyzing the MCC tree of HCV 3a, that there was a drug trafficking route linking Xinjiang and Pakistan, and proposed another possibility that a precursor HIV subtype C strain of CRF07_BC may have originated from the “Golden Crescent”, the second largest heroin production area at the border of Afghanistan and Pakistan^29^, and was introduced to IDUs in Xinjiang before recombining with subtype B’ strain to form CRF07_BC. However, this argument was based on a small number of short subtype C sequences [HXB2: 7095–7328 nt (234-bp)], and the analysis did not include the HIV sequences from Pakistan^12^. In the present study, we used all available 140 NFLG sequences of B’/C recombinants from China and Myanmar (No B’/C recombinant NFLG sequence from other countries bordering on China can be accessed from the Los Alamos HIV sequence database). The topology structures of the phylogenetic trees and tMRCA estimation of the lineages from different selected gene regions (Ia + Ib, II, and III) were highly consistent. More convincingly, the discrete phylogeographic and spatial diffusion analysis provide a clear temporal dynamic vision that both CRF07_BC and CRF08 BC came from western Yunnan in the early-1990s.

In conclusion, CRF07_BC and CRF08_BC were generated from a setting of intermixing of subtype B’, subtype C, and their recombinants, with many cycles of coinfection under some extrinsic and/or essential factors, that are still unknown, led to fixation of particular recombinant forms in populations. There are unique demographic, emigrational and cultural conditions in the border region of western Yunnan, which facilitate the dynamic HIV-1 recombination events even until today^30^. It is worthwhile to further study this phenomenon in western Yunnan to monitor and prevent the spread of new CRF07_BC or CRF08 BC like viruses.

### More precise breakpoint analysis approaches will help to evaluate the evolutionary relationships between HIV recombinant strains

In this study we used a group of reference sequences rather than a single sequence as the parental reference for subtypes B and C (B = 45 and C = 37, see methods) in the identification of recombinant breakpoints. This strategy greatly emphasized the characteristics of subtypes, and reduced the effects of random point mutations on breakpoint identification. The breakpoints of all the CRFs_BC and URFs_BC used in this study were redefined with Simplot 3.2 by comparing with preset groups of subtype B’ and C references. The results revealed that aside from the breakpoints identical with those described in previous studies, there are many new breakpoints, mostly located at both ends of very short recombinant fragments (between 50–100 bp). Short recombination fragments will be ignored if the window size is set as 300 or higher when using bootscaning or similarity plot analysis. But they can be found when the window size is set as 200, 100, or even lower. This may be one of the reasons that the mosaic structures of some CRFs have not been accurately defined. Wrong parental sequences selection could be another possible reason. Setting of a smaller windows size (200, 100, or even lower) may lead to more false positives and noise for identification of breakpoints, but provide more opportunities to obtain the information of shorter recombinant fragments. The limitation for this study is we cannot provide a much more sensitive method to define these recombination fragments. The results need be adjusted by checking the effective informative sites between subtypes around the breakpoints. These findings suggest that at the methodological level, appropriate parental reference sequences selection and more sensitive methods are needed to accurately determine the breakpoints, especially those at the ends of short recombinant fragments in the HIV-1 genome. These breakpoints may be very important for explaining the mechanism of HIV recombination in principle and observing the relationships between different recombinant strains.

Many evidences from our results provide compelling support for western Yunnan as the geographic origin of CRF07_BC and CRF08_BC, which arose from a swarm of URFs_BC strains generated in that area in the early 1990s. We also provided a new systematic approach to explore the evolutionary relationship of complex sets of recombinants and their timeline of emergence. This could also be applied to studies aiming to explore and define the evolutionary pathway and the ancestor-progenitor relationship of a variety of pathogens^6^,^18^,^31^.

## Methods

### Samples screening of early B’/C recombinants and sequence analysis

To increase the early B’/C recombinant samples from western Yunnan, a total of 100 archival HIV-1 positive plasma specimens collected in 1996 (*n* = 76) and 1998 (*n* = 24) from consenting IDUs in Ruili county, Dehong prefecture, were used for subtype screening. According to the results of genotyping for *env* C2V3 regions, selected specimens were implemented to the near full-length genome (NFLG) sequence (HXB2: 552–9636 nt) amplification following the methods described by Li *et al.*^4^ and Rousseau *et al.*^32^. Successfully amplified HIV-1 NFLG sequences were screened with HIV BLAST (http://www.hiv.lanl.gov/content/sequence/BASIC_ BLAST/basic_ blast.html)^33^ and subsequently confirmed by the neighbor-joining tree analysis with MEGA 6.0. In addition, we determined 16 NFLG sequences of CRF07_BC (n = 11) and CRF08_BC (n = 5) strains sampled in 2006–2010 across China to increase the number of CRF07_BC and CRF08_BC reference sequences in order to improve the reliability of the analyses. The NFLG sequences determined in this study are available in GenBank under accession numbers KF250366- KF250385. Informed written consent was obtained from all participants. The methods were carried out in accordance with the approved guidelines. All experimental protocols were approved by the institutional review boards of the National Center for AIDS/STD Control and Prevention, China CDC.

### NFLG sequences collection and sequence alignment

A total of 140 NFLG sequences of B’/C recombinant strains with known sampling years, including CRF07_BC (n = 34), CRF08_BC (n = 33), CRF57_BC (n = 5), CRF61_BC (n = 4), CRF62_BC (n = 3), CRF64_BC (n = 5), and URFs_BC (n = 56, 54 from China and 2 from Myanmar) were used in this study. Sequences except those newly obtained from this study were from the Los Alamos HIV Sequence Database (http://www.hiv.lanl.gov, accessed in Feb 2015 (see also Table 2). In addition, the representative NLFG sequences of non-recombinant forms of subtypes B (n = 45) and C (n = 37) from various regions in the world were chosen and retrieved from the Los Alamos HIV Sequence Database as references for the recombination breakpoints and phylogenetic analyses. An initial alignment was performed using Gene Cutter from the Los Alamos HIV sequence database (http://www.hiv.lanl.gov/content/sequence/GENE_ CUTTER/cutter.html), and then the alignment was manually adjusted using BioEdit v7.0.9^34^

### Precise recombination breakpoint analysis

The NFLG sequences of B’/C recombinants were screened and analyzed to detect recombination using the jumping profile Hidden Markov Model (jpHMM)^35^. Boot scanning and similarity plot analyses implemented in Simplot 3.2^27^ were used to define their recombinant breakpoints. NFLG sequences of subtype B’ (n = 45) and subtype C (n = 37) strains were grouped and used as the respective group of reference sequences for the precise recombinant breakpoint analyses by referring to the method of McCutchan *et al.*^7^. From a total of 140 B’/C recombinant strains, NFLG sequences of CRFs_BC including CRF07_BC (n = 34), CRF08_BC (n = 33), CRF57_BC (n = 5), CRF61_BC (n = 4), CRF62_BC (n = 3), CRF64_BC (n = 5), URFs_BC from Myanmar (n = 2, designated URFs_BC_MM_), and 7 newly determined NFLG sequences of URFs_BC from the archival specimens derived from Dehong prefecture of western Yunnan in 1996–1998 (designated URFs_BC_YN_) were selected. The URFs_BC identified later than 2000 from China were not implemented in the comparison of recombination breakpoints for the reason that the sampling time of these strains may be too far from the time when CRF07_BC and CRF08_BC were generated. Many cycles of co-infection subtypes and CRFs may lead to confusing results. Boot scanning analyses were performed with 2 different windows and step sizes (window = 200 nt, step = 20 nt; window = 100 nt, step = 10 nt) to define the precise subtype structure of each recombinant and were compared with similarity plot analysis by window size 100 and step size 10 (Supplemental Figure S2). Effective informative sites between subtypes around each breakpoint found above were carefully checked to determine the precise recombinant breakpoints of these B’/C recombinant forms (Supplemental Figure S3).

### Bayesian phylogenetic and molecular clock analyses

To investigate the phylogenetic and evolutionary relationship of B’/C recombinant strains in this study, Bayesian phylogenies for each well-chosen subtype C region were inferred under the GTR + γ4 substitution model using Markov Chain Monte Carlo (MCMC) sampling, as implemented in BEAST v.1.7.4 using an uncorrelated log-normal relaxed molecular clock model^36^. A Bayesian skyline plot coalescent model with 500 million steps was used to estimate the evolutionary rate and the divergence times to the most recent common ancestor (tMRCA) of the respective subtype C and its recombinant lineages. The first 10% states of each run were discarded as burn-in to produce at least 450 million effective states in order to estimate evolutionary rate and the tMRCA of various lineage C segments. Convergence was checked using Tracer v1.5 (http://beast.bio.ed.ac.uk/Tracer), which made sure that all parameters had effective sample sizes >200. The maximum clade credibility (MCC) trees were visualized using the program FigTree v1.4.0 (http://beast.bio.ed.ac.uk) with the same calculation.

### Discrete phylogeographic analyses and spatial structure

The location factor of discrete, phylogeographic analyses was used to identify the geographic origin and the diffusion pattern of HIV-1 B’/C recombinant strains in China. The exchange process of locations throughout the entire phylogeny was modeled using symmetric continuous-time Markov chains (CTMCs)^37^ with an approximate CTMC conditional reference prior on the overall scalar rate. Bayesian stochastic search variable selection (BSSVS) procedures were used to infer a minimum set of location exchange rates that provided an adequate description of the process of viral dissemination^37^. These procedures offer a Bayesian factor (BF) test to identify the most adequate parsimonious description of the process of spatial spread. KML files generated by software SPREAD (Spatial Phylogenetic Reconstruction of Evolutionary Dynamics)^38^ were used to visualize the output from Bayesian phylogeographic analysis. A sketch map was drawn referring to the Bayesian inference of diffusion process which can be displayed follow the software instructions of SPREAD (http://www.kuleuven.be/aidslab/phylogeography/SPREAD.html).

## Additional Information

**How to cite this article**: Feng, Y. *et al.* Geographic origin and evolutionary history of China's two predominant HIV-1 circulating recombinant forms, CRF07_BC and CRF08_BC. *Sci. Rep.* **6**, 19279; doi: 10.1038/srep19279 (2016).

## Supplementary Material

Supplementary Table S1, Figure S1-S4

Supplementary Dataset 1

## Figures and Tables

**Table 1:** Summary of the shared recombination breakpoints in CRFs_BC and URFs_BC in China.

HIV-1 genotype	Strain (Reference) name	Location of Strains or reference strains [City/District (Region)]	Shared recombination breakpoints^a^								Number of recombination breakpoints shared with CRF07_BC and/or CRF08_BC
			*gag*		*pol*	*vpr*	*tat*	*env*	*nef*		
			Site1	Site2	Site3	Site4	Site5	Site6	Site7	Site8	
			1215	1641	3180	5694	6045	6431	8873	9059	
CRF07_BC	97CN54	Xinjiang	+	+	+	+	+	+	+	+	**8**
CRF08_BC	97CNGX_6F	Guangxi	+	+	+				+	+	**5**
CRF57_BC	341	Baoshan (Western Yunnan)	+			+		+			3
CRF61_BC	JL100010	Jilin	+		+						2
CRF62_BC	YNFL13	Ruili/Dehong (Western Yunnan)					+	+			3
CRF64_BC	YNFL31	Ruili/Dehong (Western Yunnan)									3
URFs_BC before 2000	96CN.YNRL9607	Ruili/Dehong (Western Yunnan)									0
	96CN.YNRL9613	Ruili/Dehong (Western Yunnan)	+		+						2
	96CN.YNRL9618	Ruili/Dehong (Western Yunnan)				+			+		3
	96CN.YNRL9828	Ruili/Dehong (Western Yunnan)			+						1
	96CN.YN3018^b^	Ruili/Dehong (Western Yunnan)				+			+	+	4
	96CN.YN4007	Ruili/Dehong (Western Yunnan)									0
	96CN.YN4018	Ruili/Dehong (Western Yunnan)							+	+	3
**Numbers of recombination breakpoints of CRF07**_**BC and CRF08**_**BC shared in Yunnan URFs**			3	1	3	4	4	2	4	3	24

^a^The sites of recombination breakpoints refer to reference sequence HXB2.

^b^The genome region from 1-2462 nt of YNRL3018 was not successfully amplified and sequenced. So the sequence of this strain is shorter than other NFLG sequences.

**Table 2:** NFLG sequences of the CRFs_BC and URFs_BC from China and Myanmar used in the study.

Strain	N	Country of origin	Year of sample collection	Study site (Province/Region) and sampling number
CRF07_BC	34 (11)	China	1997–2013	Beijing 1, Guangdong 1, Guangxi 2, Guizhou1, Hebei 2, Jiangsu 1, Liaoning 3, Ningxia 1, Sichuan 6, Taiwan 2, Xinjiang 10, Yunnan 4
CRF08_BC	33 (5)	China	1997–2007	Guangdong 2, Guangxi 6, Ningxia 1, Yunnan 22
CRF57_BC	5	China	2007–2010	Yunnan 5
CRF61_BC	4	China	2007–2010	Fujian 2, Jilin 2
CRF62_BC	3	China	2010	Yunnan 3
CRF64_BC	5	China	2009–2010	Yunnan 5
URFs_BC	54 (7)	China	1996–2010	Guangdong 1, Guangxi 1, Jiangxi 1, Jilin 1,Yunnan 50
	2	Myanmar	1999	Mandalay (Central Myanmar) 2
Total	140 (23)		1997–2013	

The numbers of NFLGs newly obtained from this study are shown in the parenthesis.
