# Supplementary material for: Geographic origin and evolutionary history of China’s two predominant HIV-1 circulating recombinant forms, CRF07_BC and CRF08_BC
Source: Sci Rep. 2016 Jan 14;6:19279. doi: 10.1038/srep19279 (PMC4725877; doi:10.1038/srep19279)

Supplementary Materials for

# Geographic origin and evolutionary history of China's two predominant HIV-1 circulating recombinant forms, CRF07_BC and CRF08_BC

Yi Feng*, Yutaka Takebe *, Huamian Wei, Xiang He , Jenny H. Hsi, Zhenpeng Li, Hui Xing[1], Yuhua Ruan, Yao Yang, Fan Li, Jing Wei, Xingguang Li, Yiming Shao

Corresponding author. Tel: +86 10 5890 0981; e-mail: yshao08@gmail.com (Y.S.)

*These authors contributed equally to this work.

**This PDF file includes:**

Supplemental Table S1

Supplemental Figure S1-S4

**Supplemental Table S1. Estimated substitution rates and tMRCAs of subtype C and its recombinant lineages in China [a]**

| HIV-1 subtype C and its recombinant lineage | tMRCA estimation [Median (95% HPD)] | | |
|---|---|---|---|
| | Region I (Ia +Ib) | Region II | Region III |
| | *gag* (HXB2:790-1191nt;1642-2010nt) | *pol* (HXB2:3331-5593nt) | *env 1* (HXB2: 6541nt-8695nt) |
| $C_{AF}$ | 1966.4 (1959.7, 1971.8) | 1968.6 (1964.5, 1972.4) | 1965.9 (1960.8, 1969.9) |
| $C_{IN}$ | 1978.0 (1972.9, 1982.9) | 1976.2 (1972.6, 1979.4) | 1971.8 (1968.4, 1974.9) |
| Yunnan URFs_BC | 1987.0 (1981.6, 1991.5) | 1988.6 (1986.4, 1990.6) | 1986.1 (1983.8, 1988.2) |
| CRF07_BC | 1994.0 (1991.9, 1995.6) | 1994.1 (1992.8, 1995.4) | 1991.6 (1989.6, 1993.3) |
| CRF08_BC | 1992.7 (1989.9, 1994.9) | 1992.7 (1990.5, 1994.4) | 1990.7 (1988.7, 1992.5) |
| CRF57_BC | | | 1993.2 (1990.7, 1995.3) |
| CRF61_BC | 2003.6 (2000.5, 2006.0) | 2002.6 (2000.1, 2004.6) | 2001.5 (1999.3, 2003.6) |
| CRF62_BC | 2003.7 (1998.8, 2007.2) | 2003.0 (2000.0, 2005.7) | 2002.3 (1999.4, 2004.9) |
| CRF64_BC | | | 1995.4 (1993.3, 1998.0) |

[a] Based on BEAST analysis under a relaxed molecular clock with GTR + γ4 substitution model and skyline model. Estimated substitution rates are $3.76\times10^{-3}$, $2.23\times10^{-3}$, and $6.36\times10^{-3}$ substitutions/site/year for *gag* (Region (Ia +Ib), *pol* (II), and *env* (III) regions, respectively. ESS values are >200 for all data sets.

Supplementary Figure S1

Neighbor-joining tree constructed with MEGA 6 using the NFLG sequences

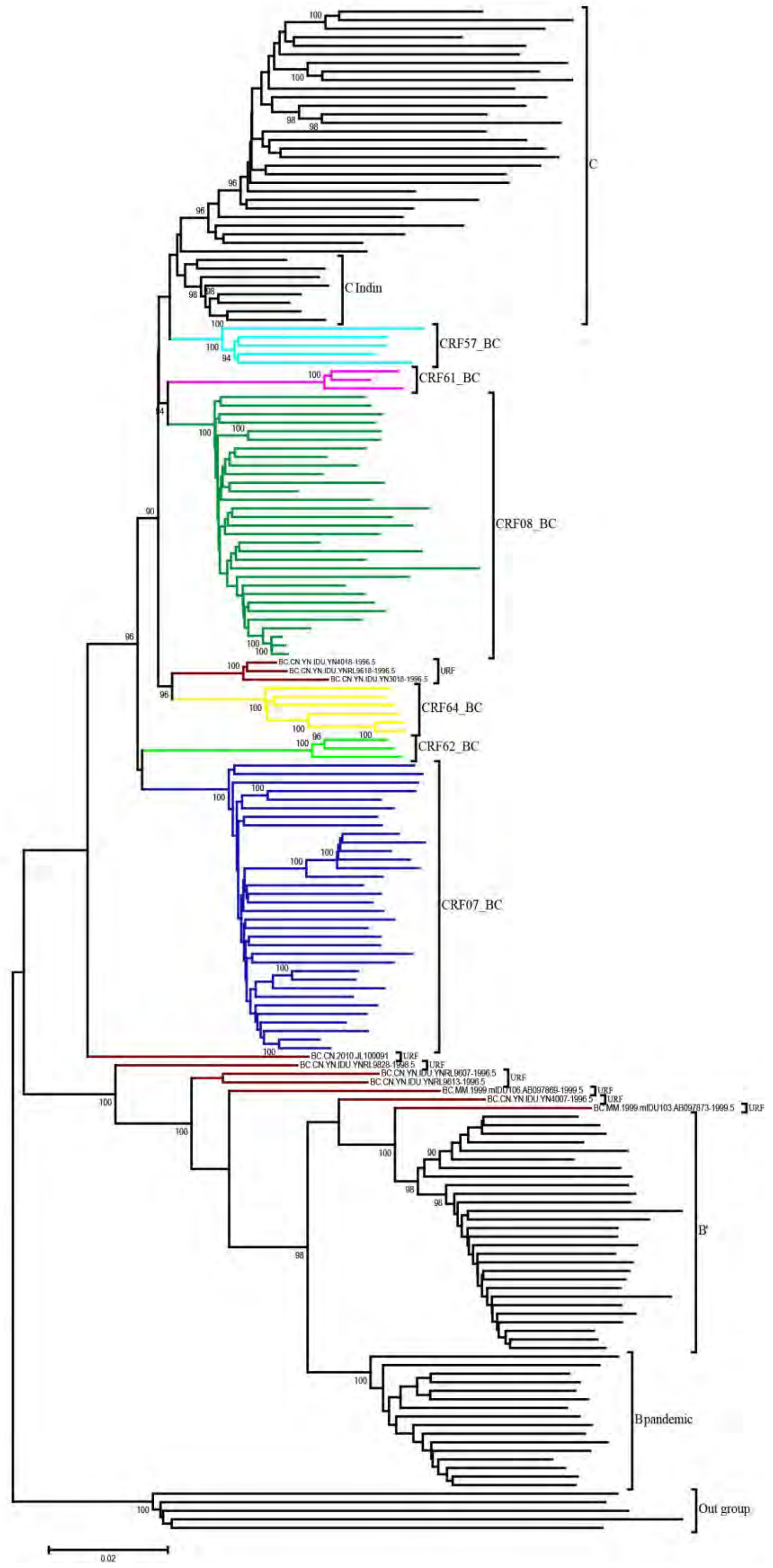

Supplementary Figure S2

Bootscanning and similarity plot analyses performed with different window and step sizes for defining the precise subtype structure of CRFs and URFs in this study

CRF07_BC

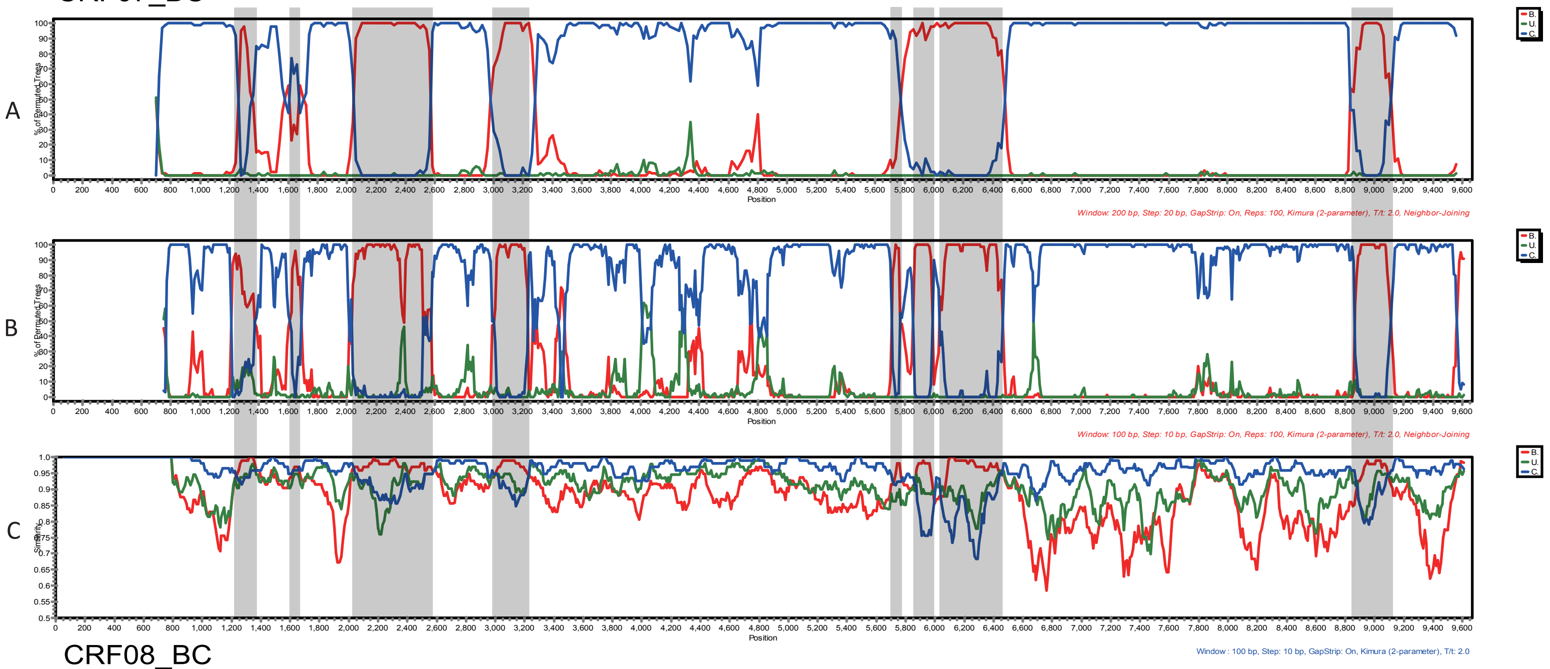


CRF08_BC

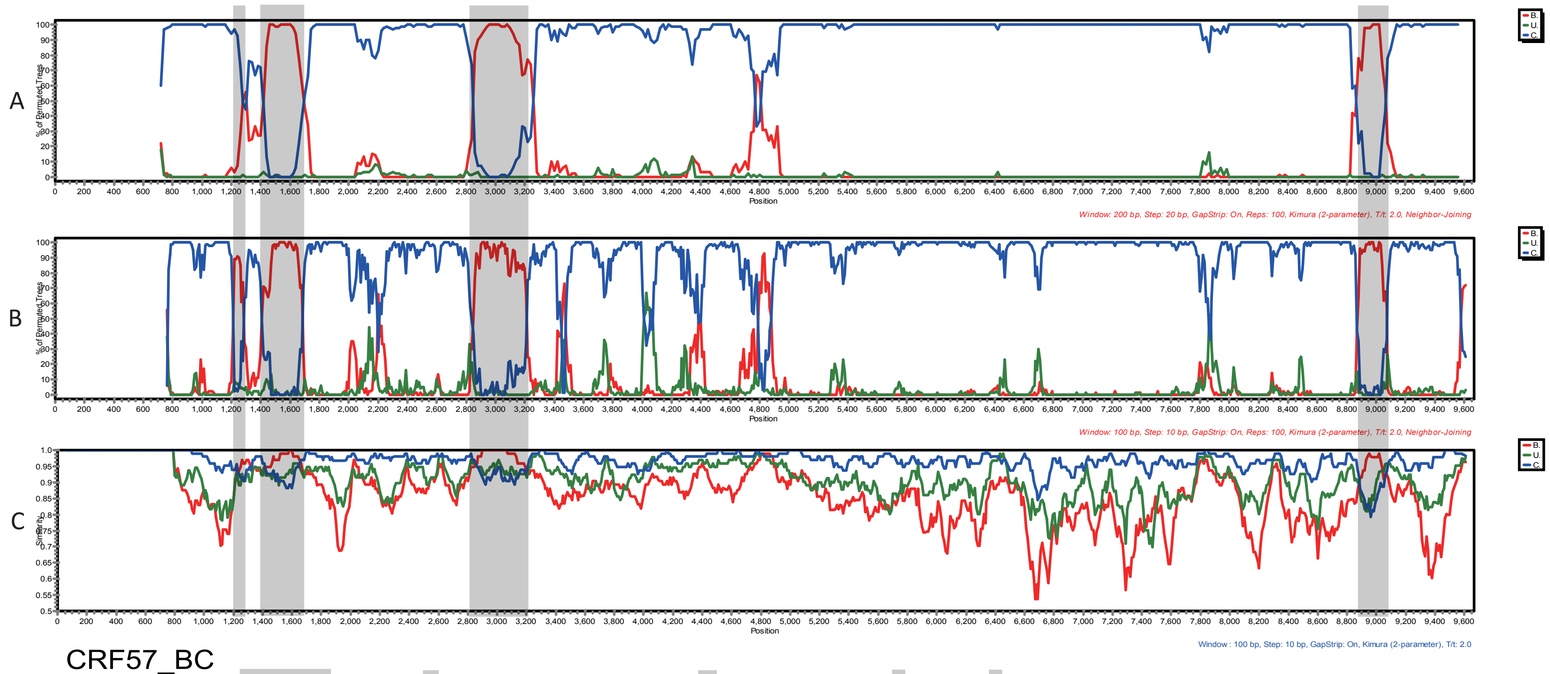


CRF57_BC

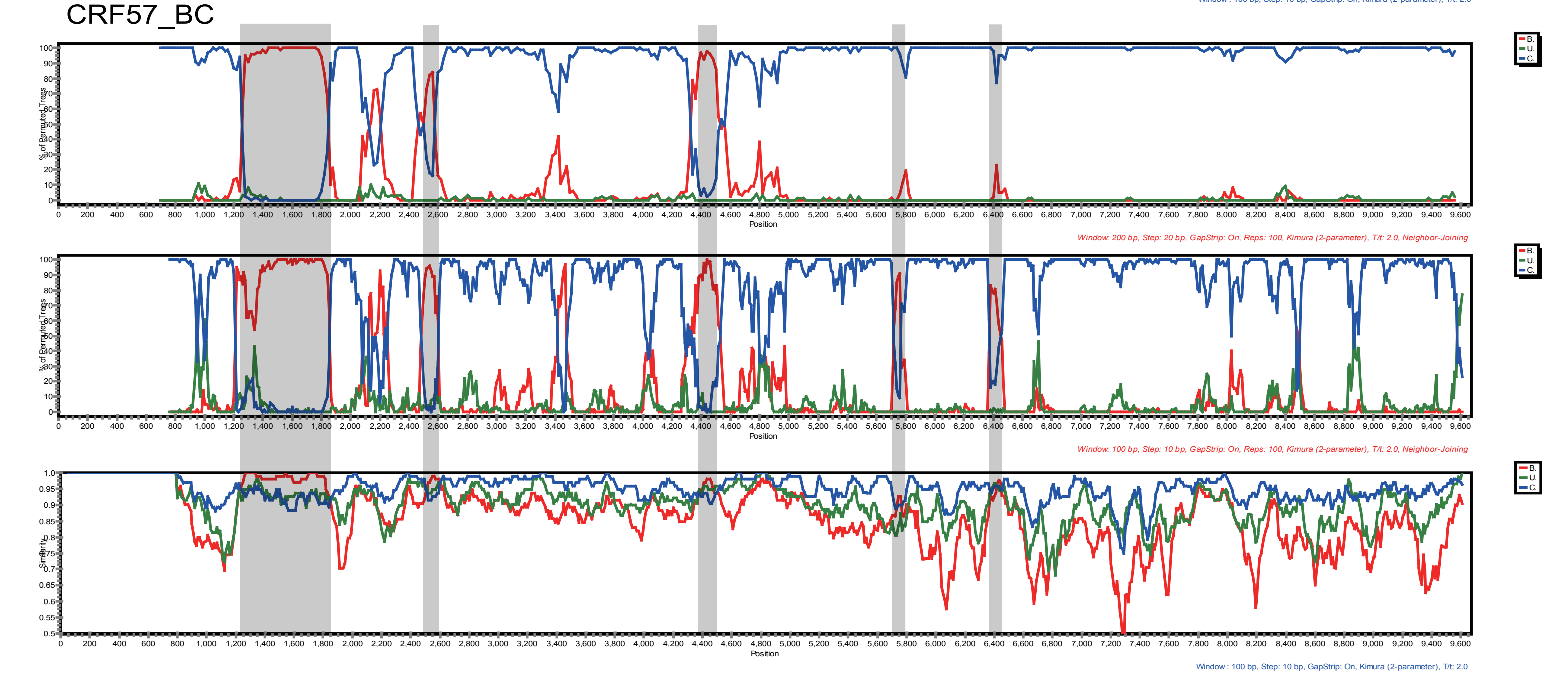

## CRF61_BC

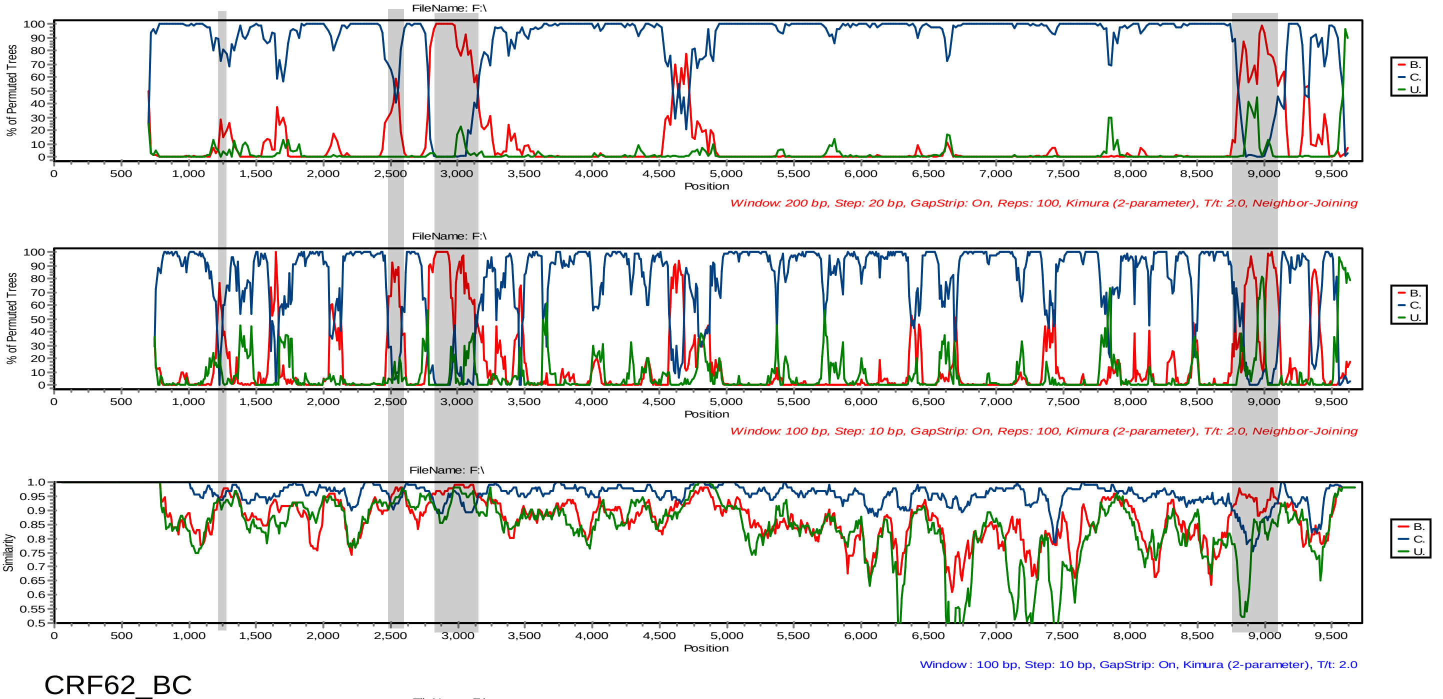


## CRF62_BC

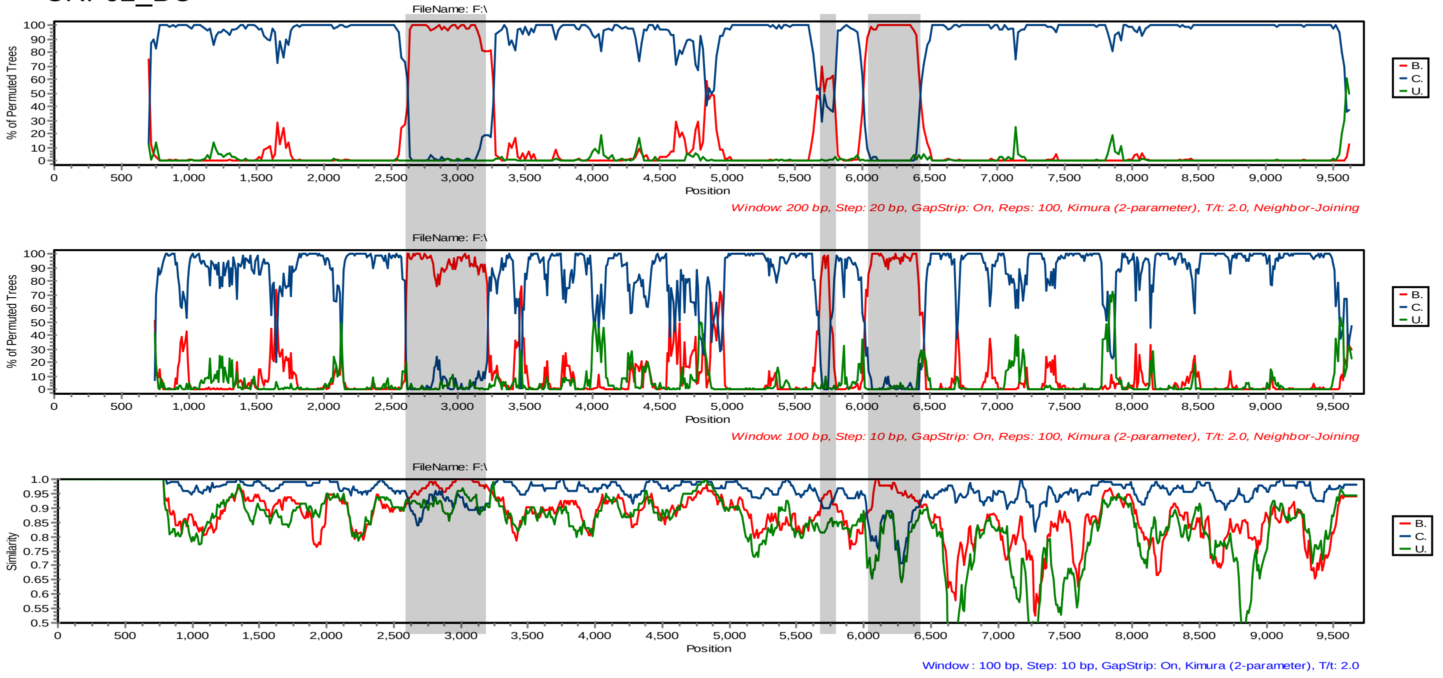


## CRF64_BC

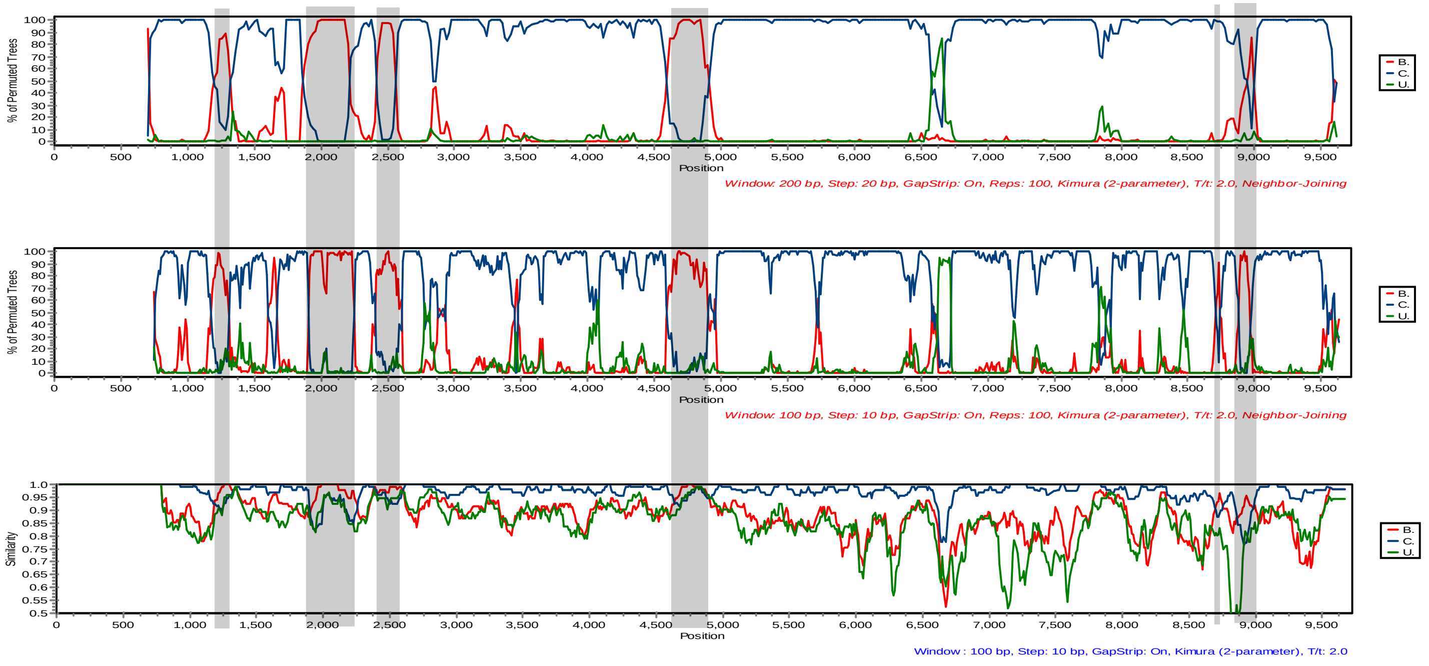

Query: YNRL9607

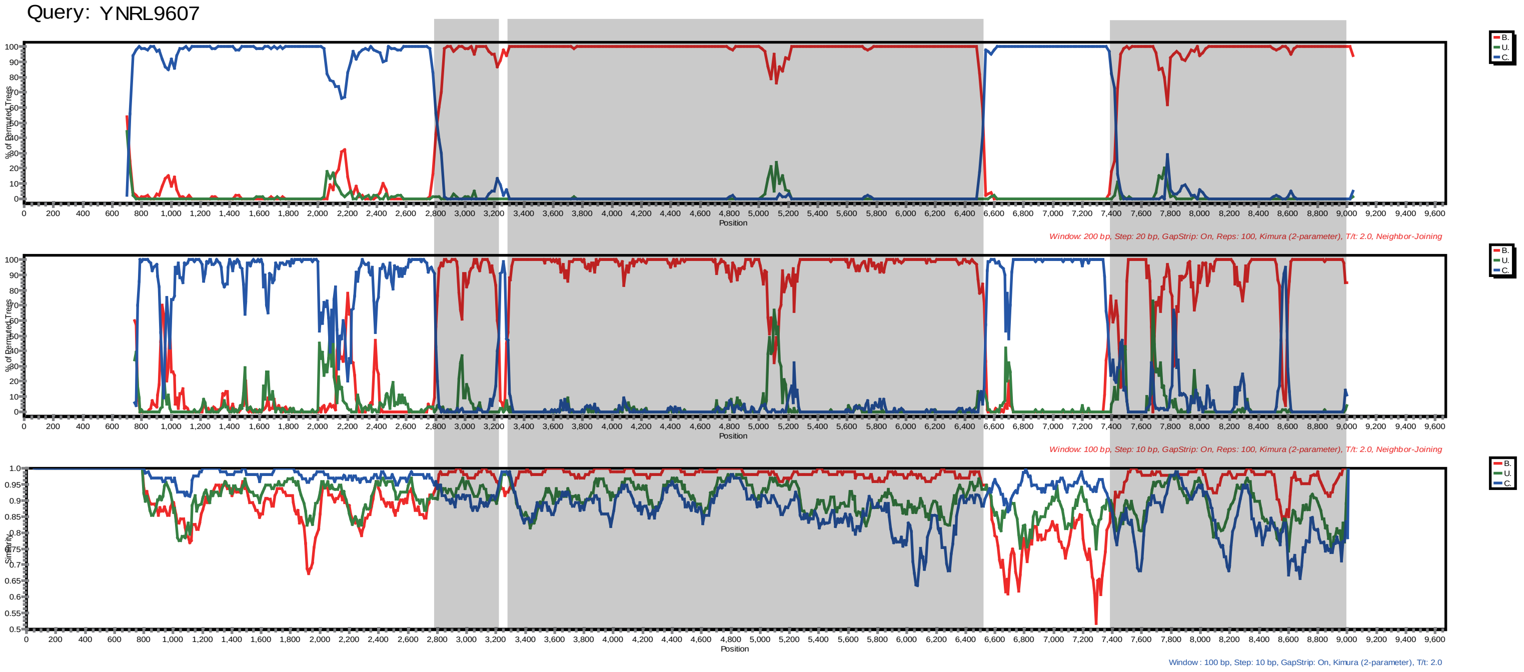


Query: YNRL9613

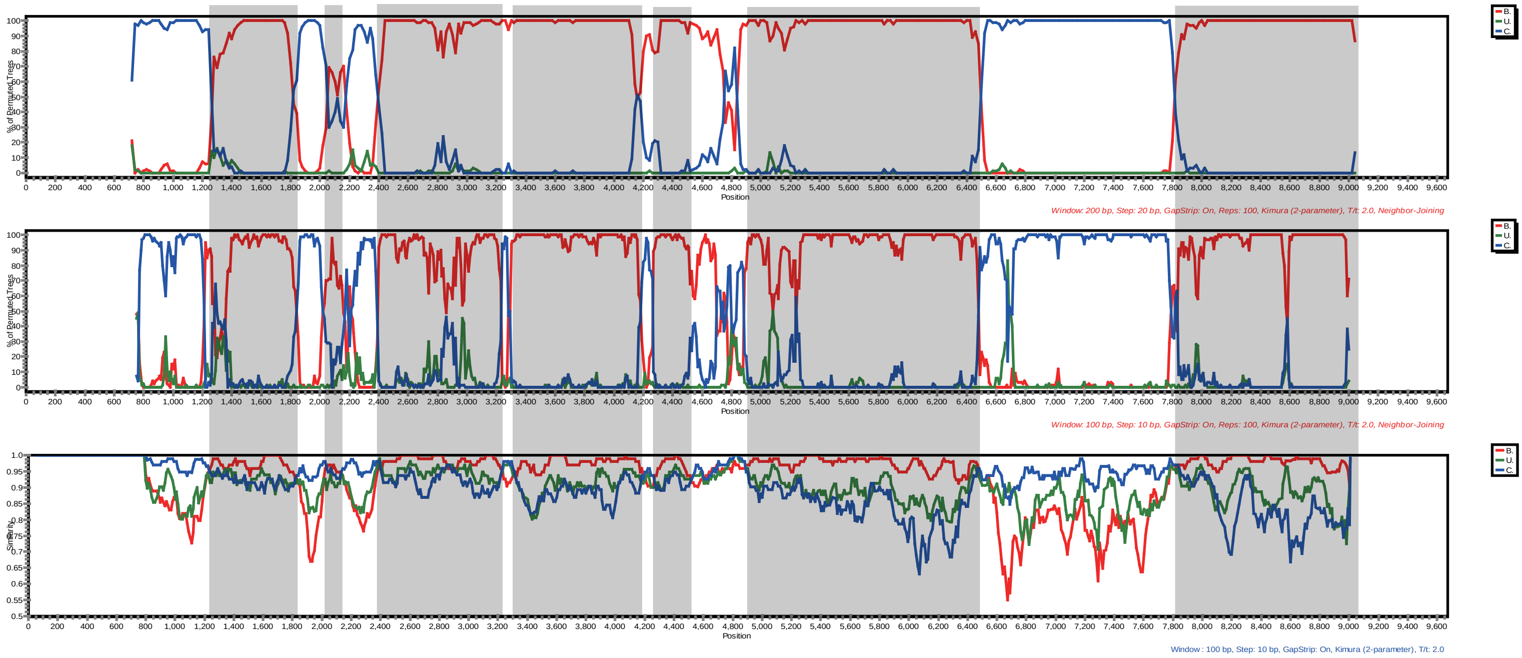


Query: YNRL9618

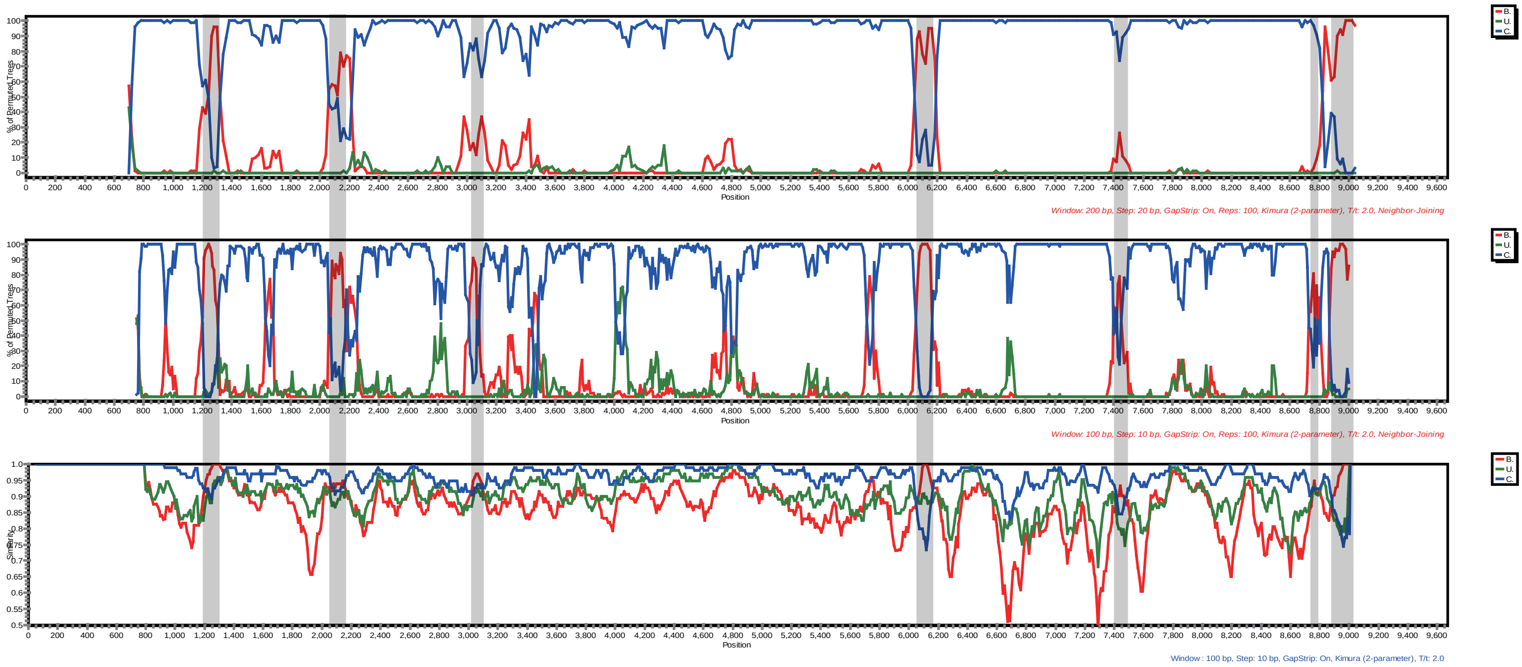

Query: YNRL9828

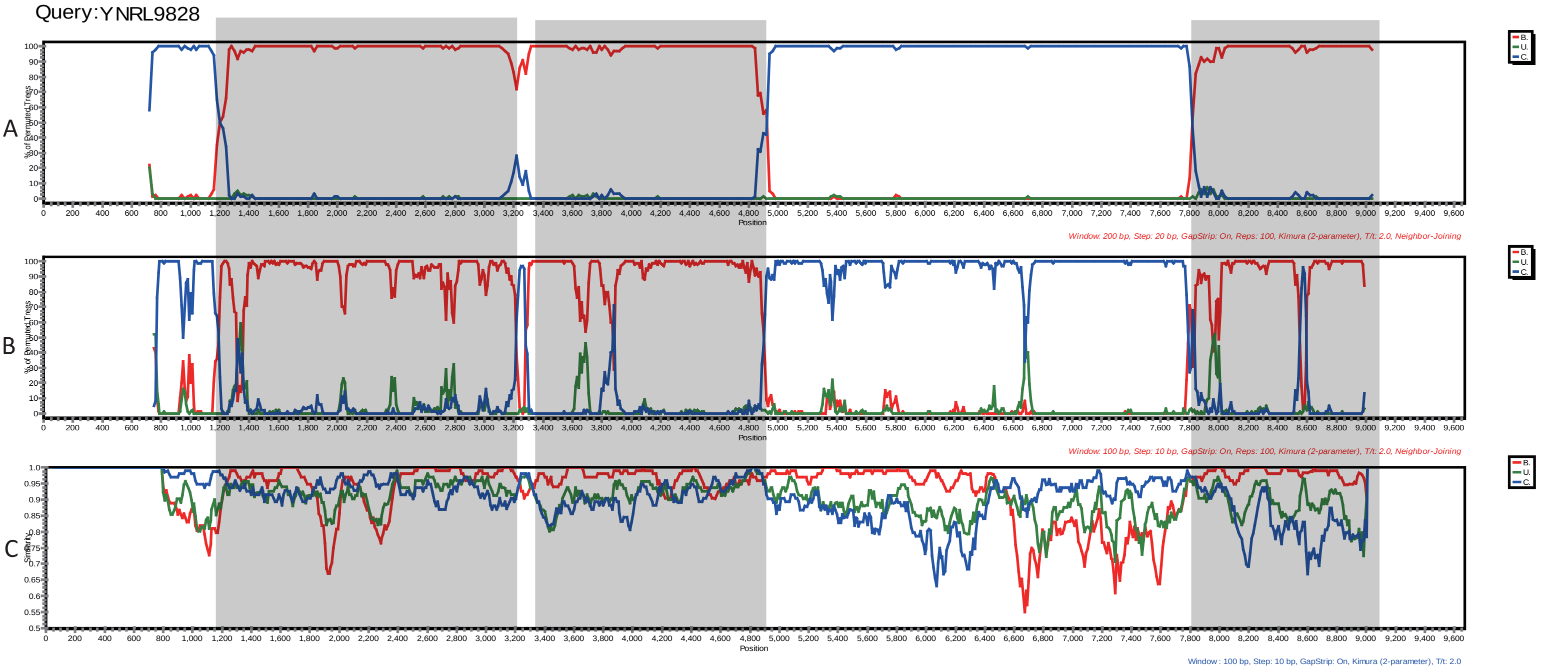


Query: YN3018

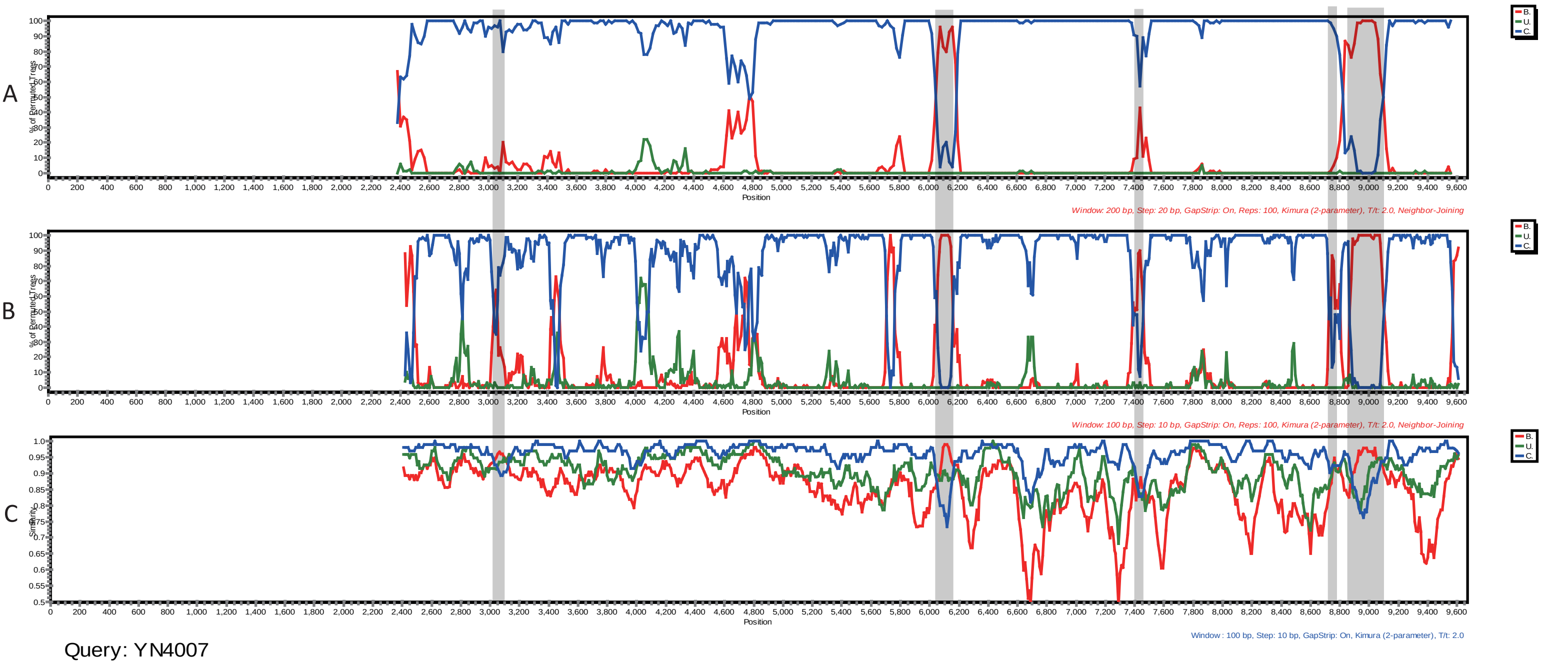


Query: YN4007

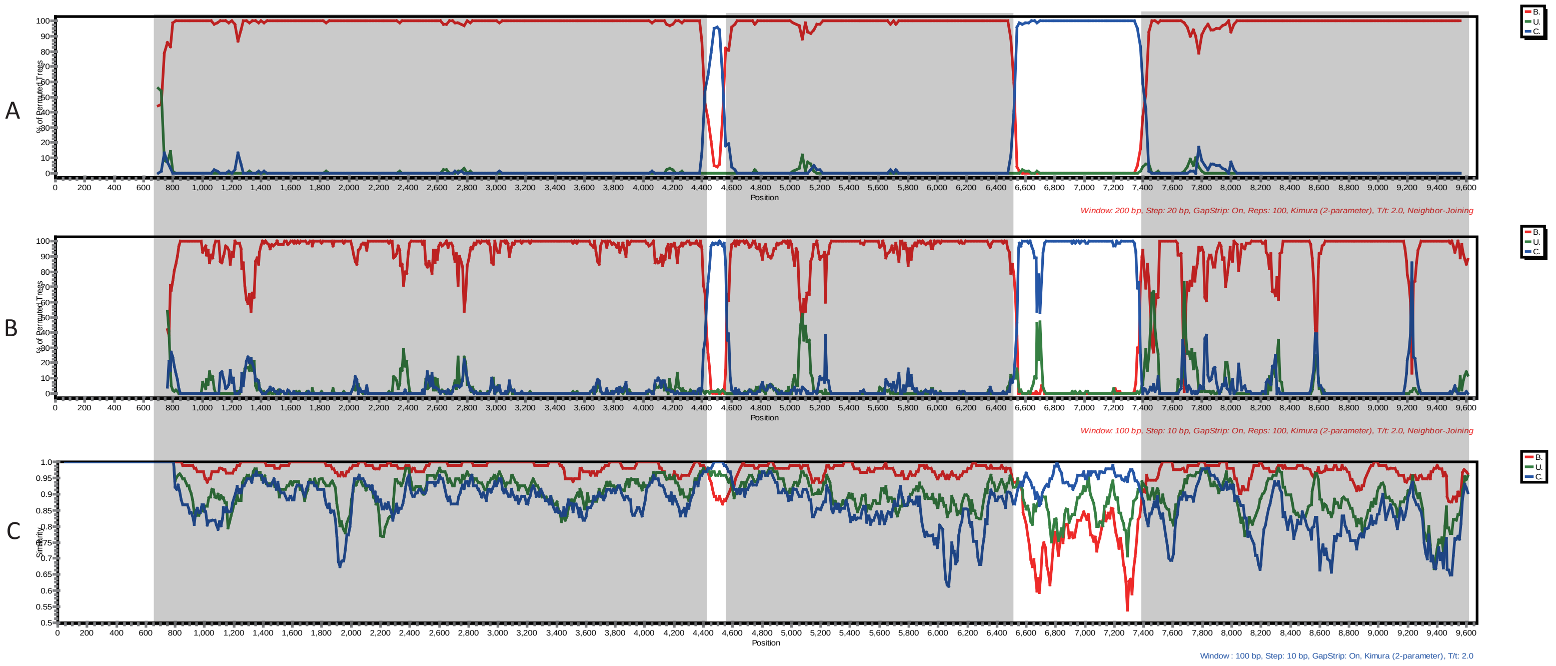

Query: YN4018

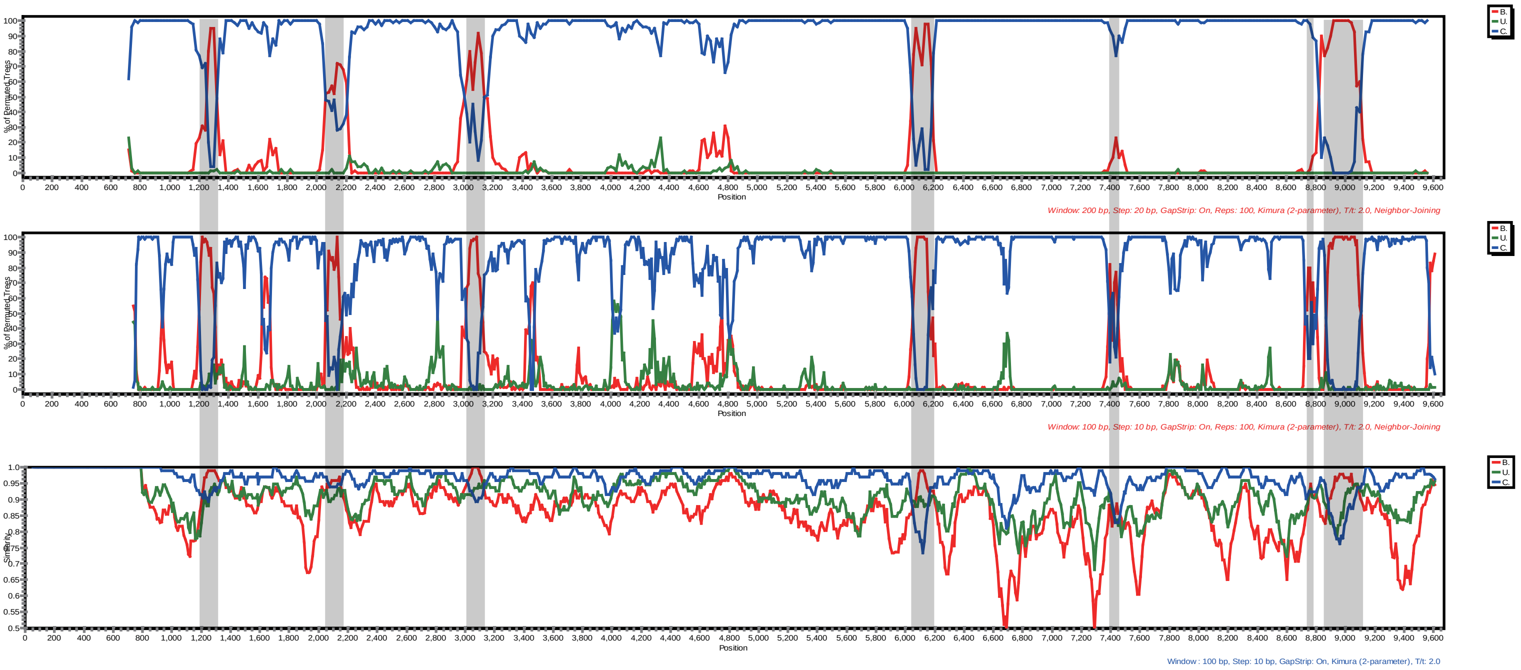


Query: mIDU103

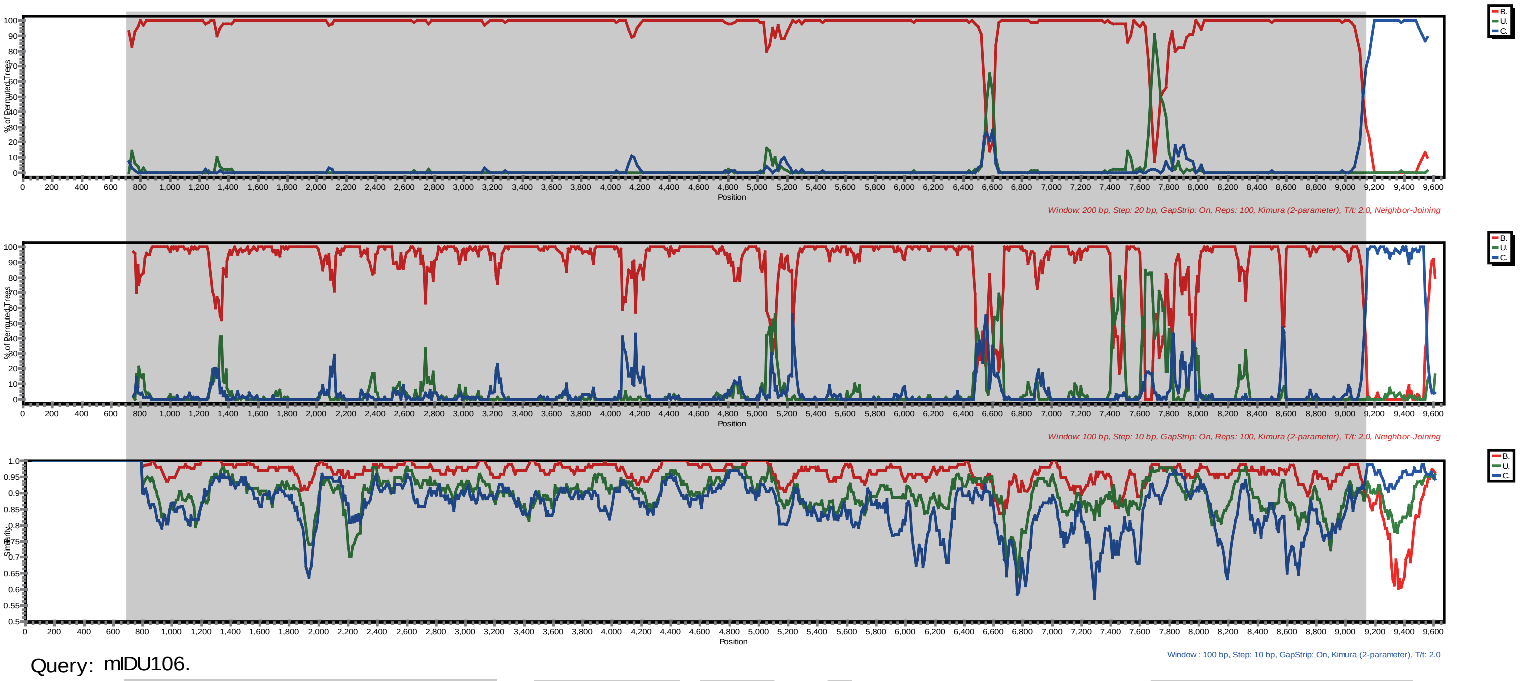


Query: mIDU106.

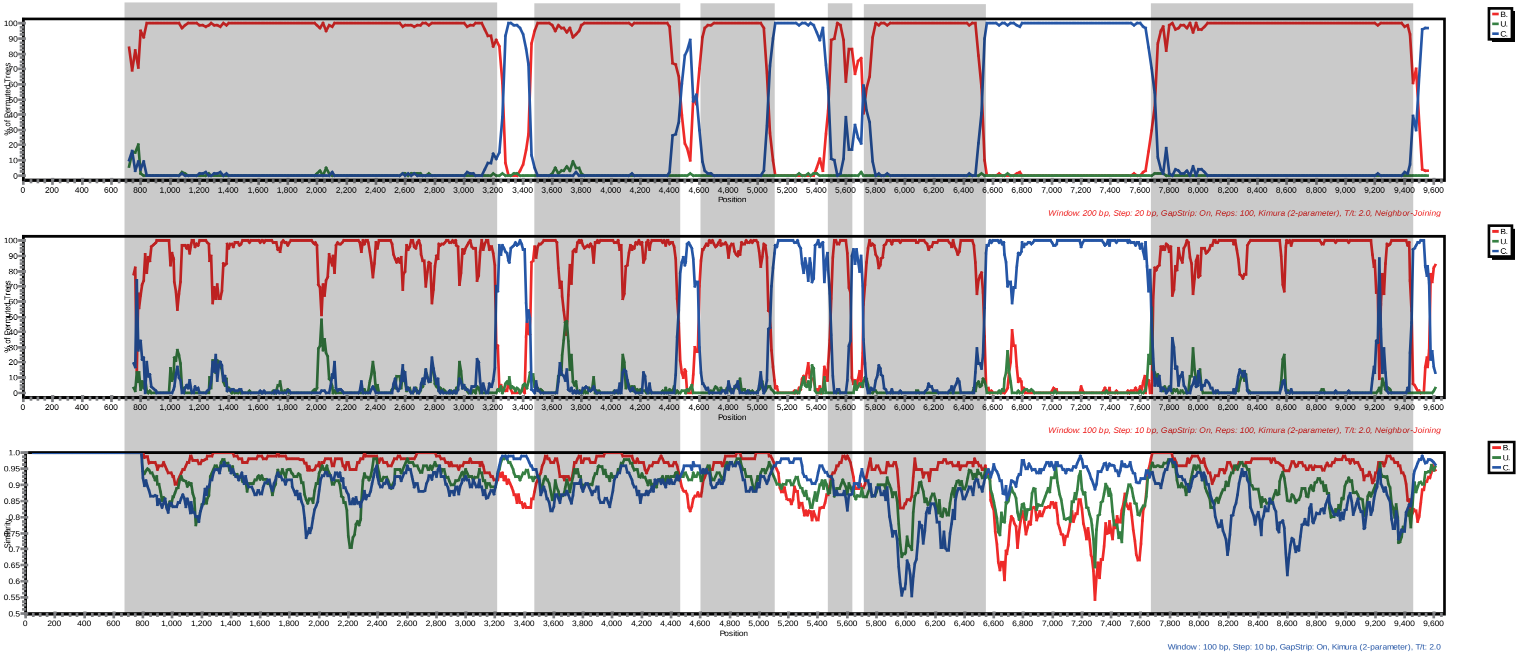

Supplementary Figure S3

The effective informative sites around the breakpoints between subtype B' and C were checked according to the results of Bootscan and Simplot. In order to show in the figure, sequences were reduced. The horizontal rectangles show the strains that share the similar informative sites around the breakpoints and the vertical rectangles show the effective informative sites around the breakpoints.

Site 1 (HXB2: ~1215)

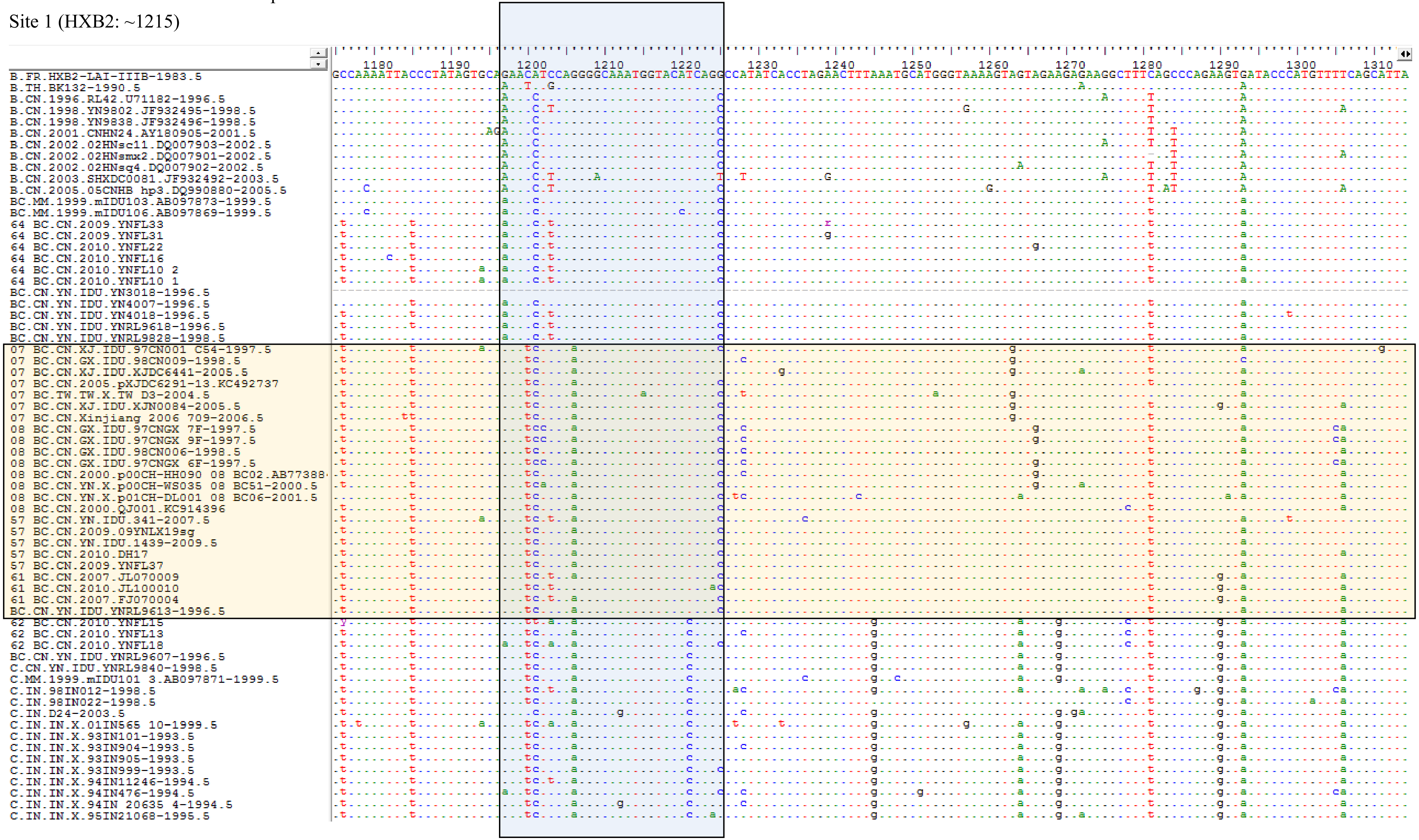

Site 2 (HXB2: ~1641)

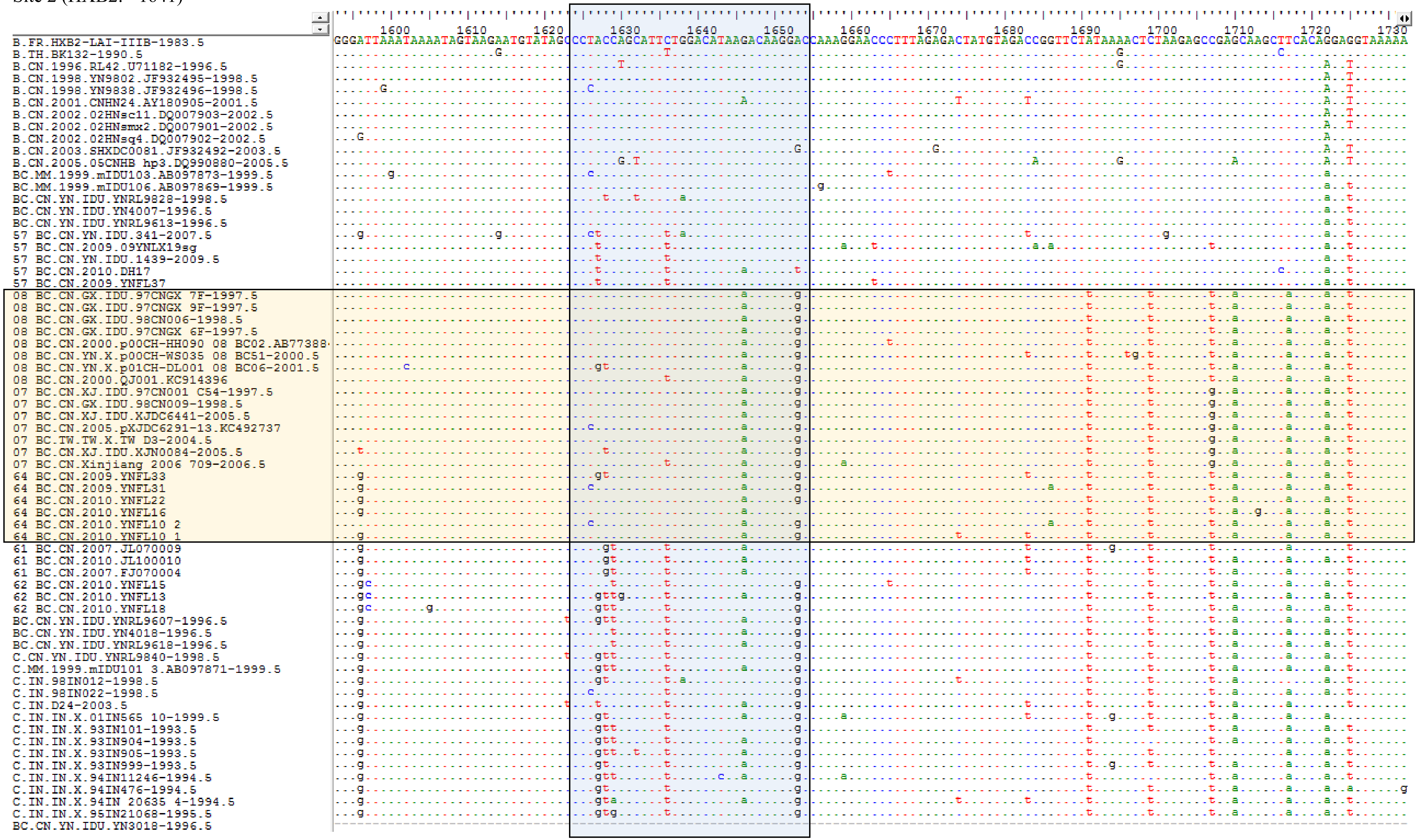

Site 3 (HXB2: ~3180)

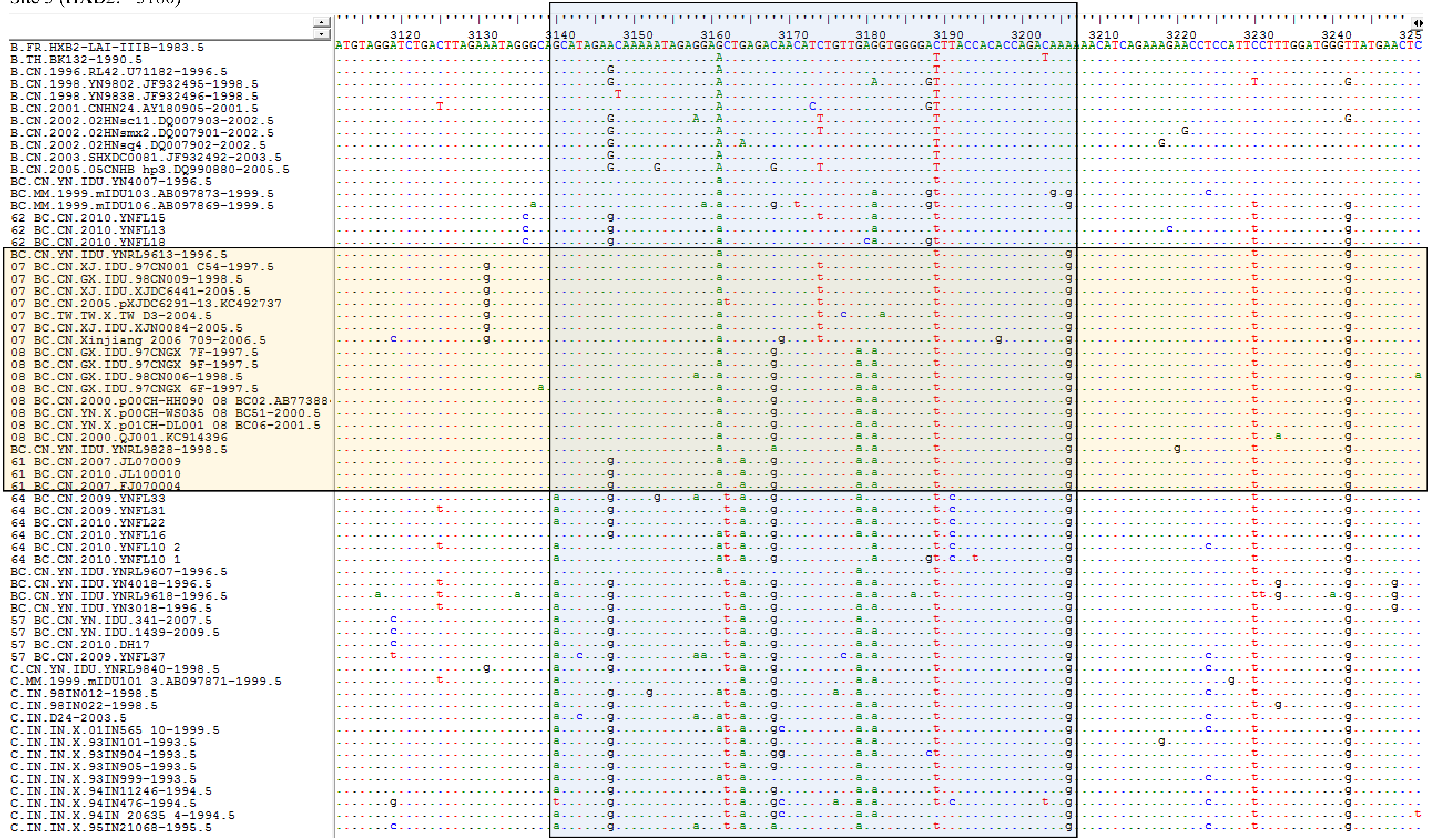

Site 4 (HXB2: ~5694)

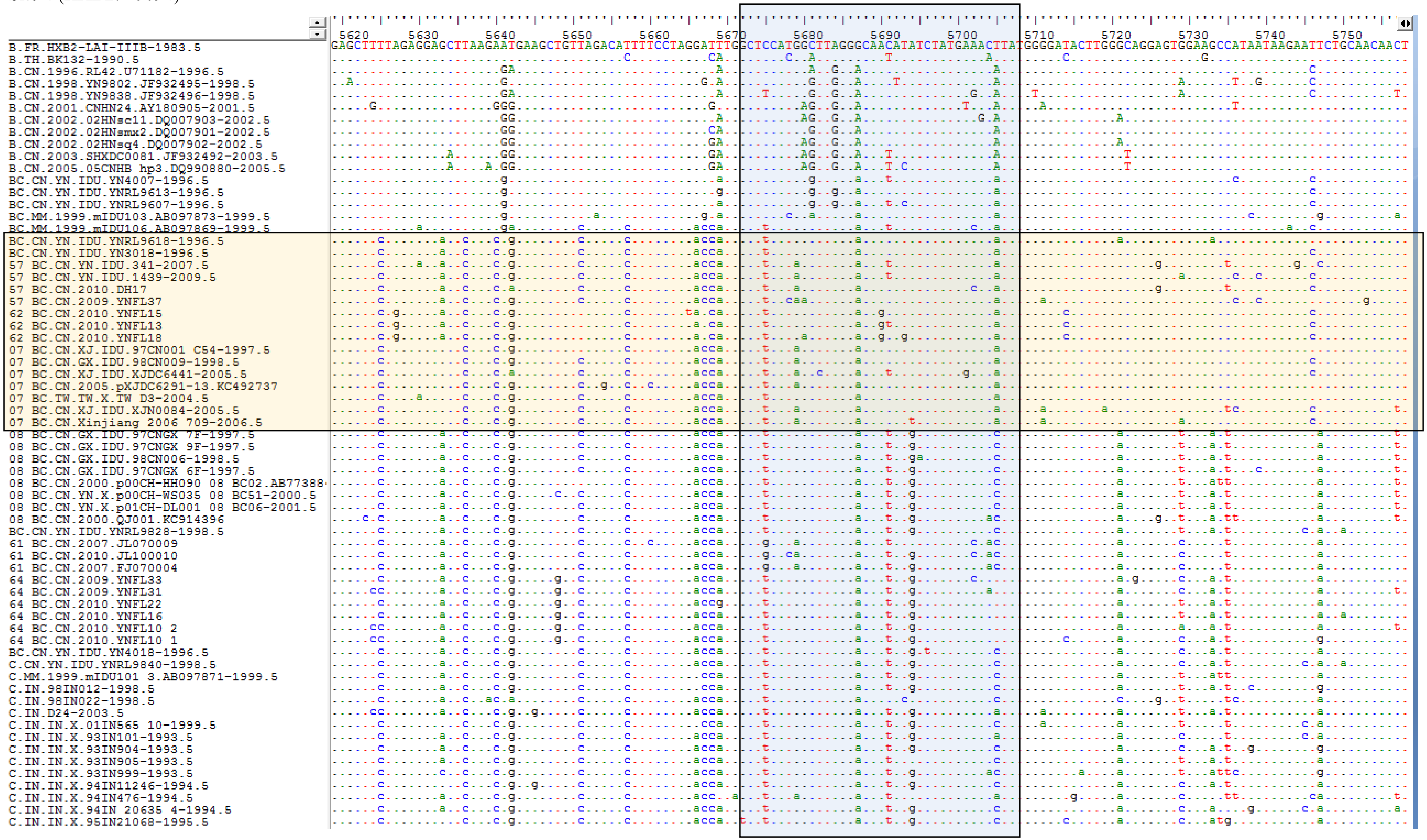

Site 5 (HXB2: ~6045)

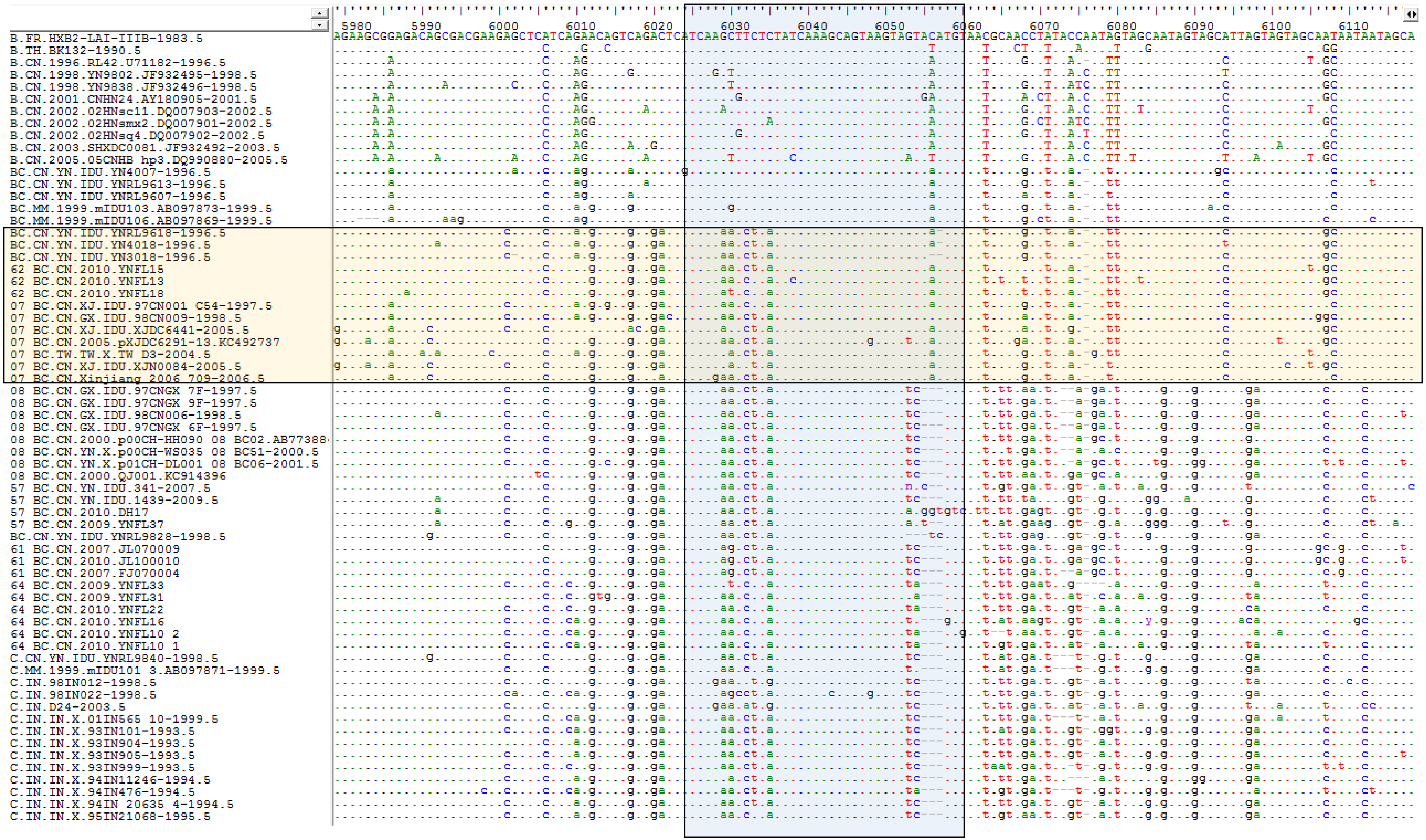

Site 6 (HXB2: ~6431)

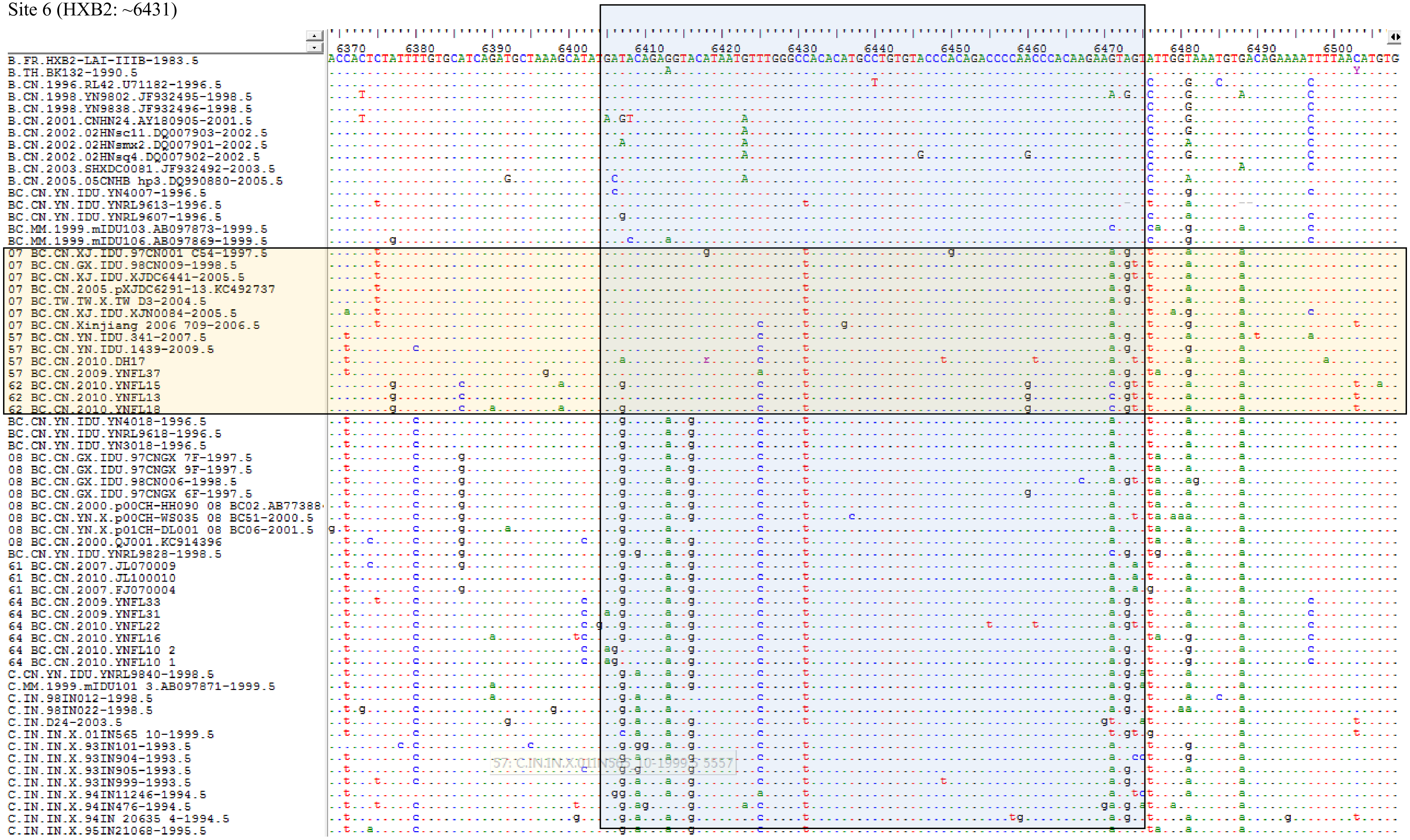

Site 7 (HXB2: ~8873)

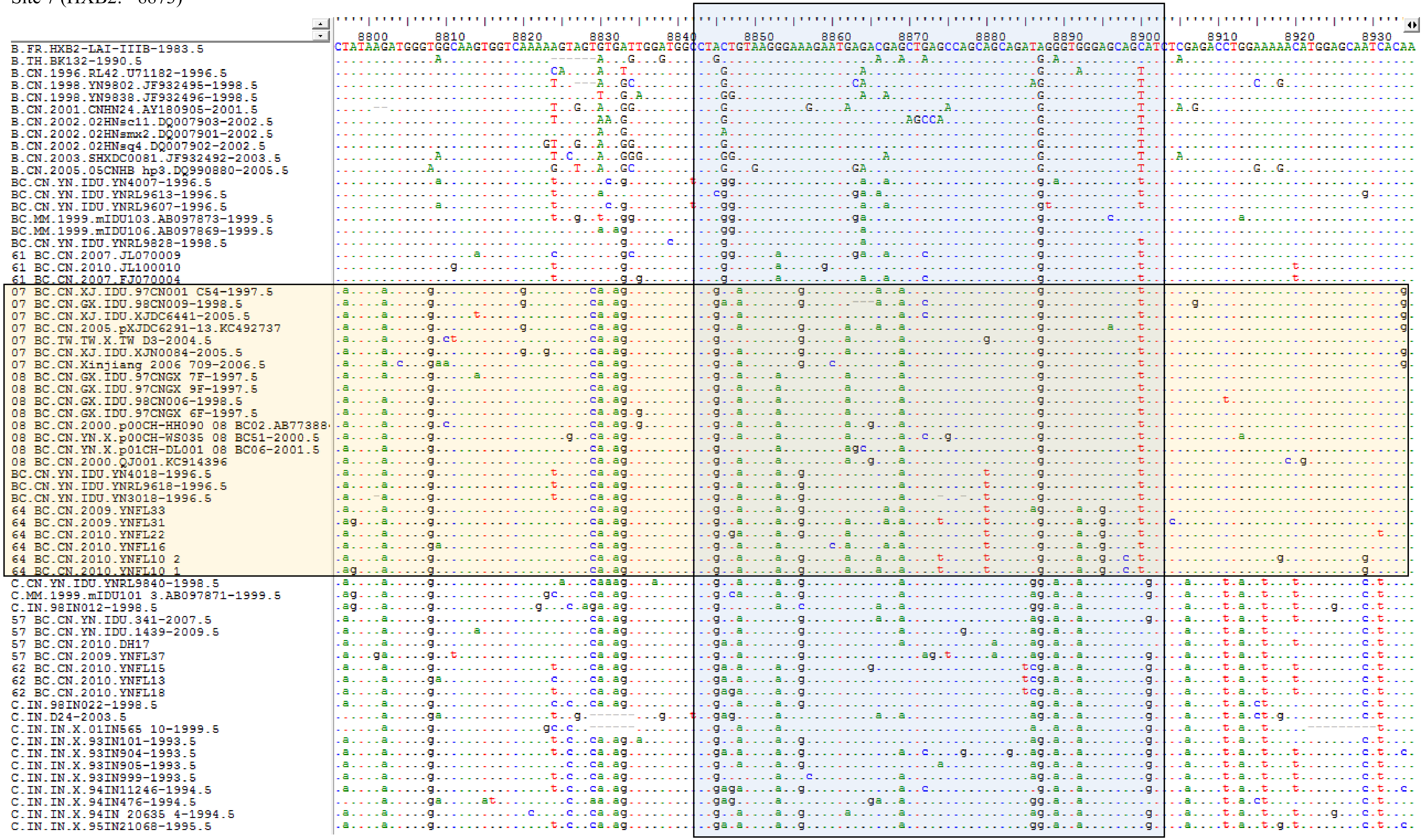

Site 8 (HXB2: ~9059)

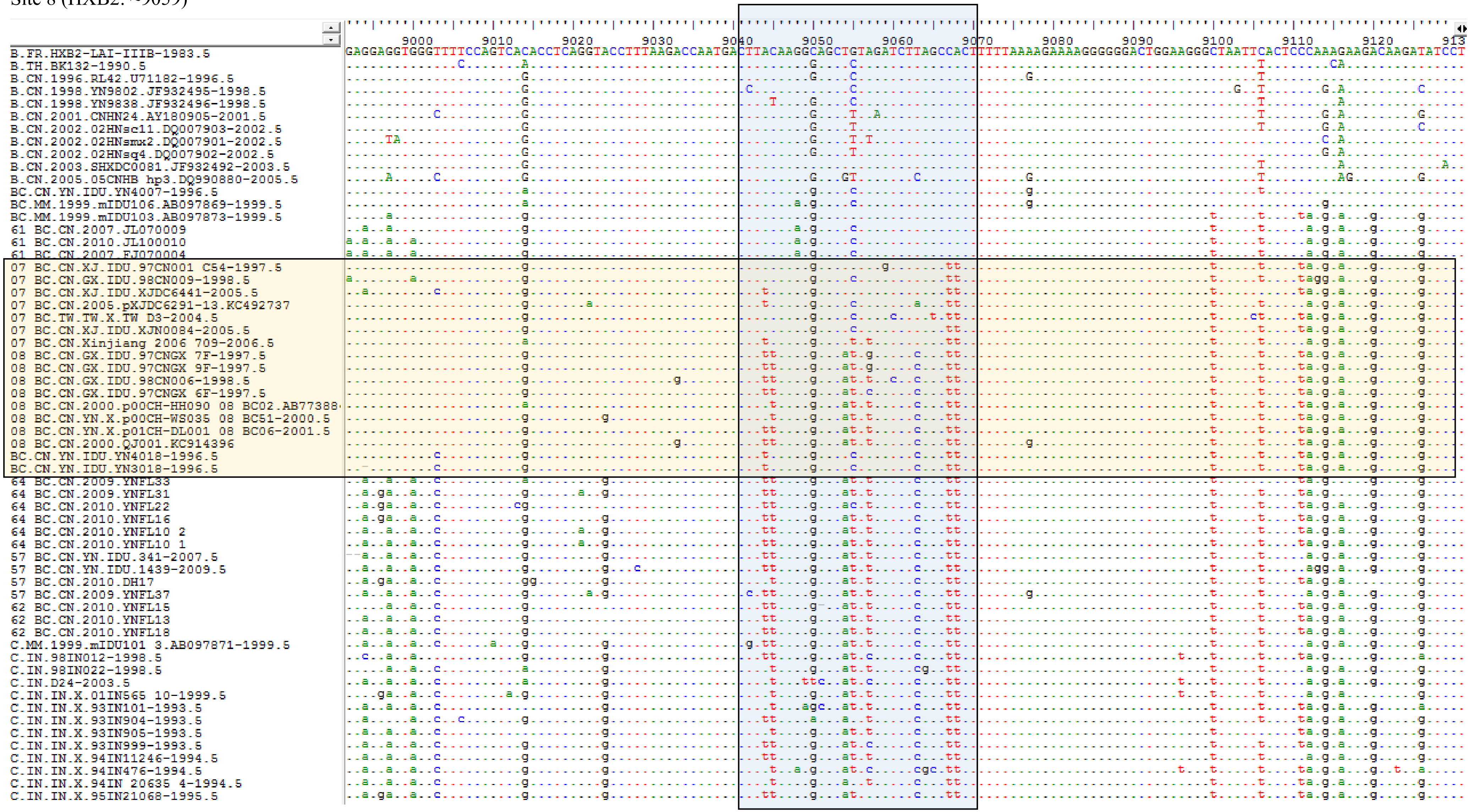

Supplemental Figure S4

The updated subtype structures and breakpoints of recombinations of the CRFs_BC

CRF07_BC

| | | |
|---|---|---|
| 1 | 789 | N |
| 790 | 1214 | C |
| 1215 | 1377 | B |
| 1378 | 1593 | C |
| 1593 | 1641 | B |
| 1642 | 2009 | C |
| 2010 | 2546 | B |
| 2547 | 2980 | C |
| 2981 | 3180 | B |
| 3181 | 5693 | C |
| 5694 | 5790 | B |
| 5791 | 5850 | C |
| 5851 | 5998 | B |
| 5999 | 6044 | C |
| 6045 | 6430 | B |
| 6431 | 8872 | C |
| 8873 | 9059 | B |
| 9059 | 9569 | C |
| 9570 | 9686 | N |

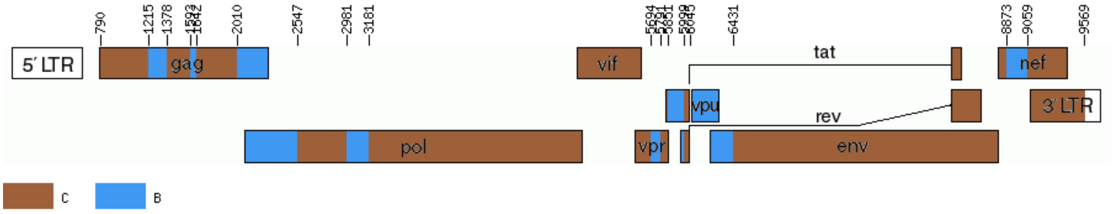


CRF08_BC

| | | |
|---|---|---|
| 1 | 789 | N |
| 790 | 1214 | C |
| 1215 | 1298 | B |
| 1299 | 1423 | C |
| 1424 | 1641 | B |
| 1642 | 2854 | C |
| 2855 | 3180 | B |

| | | |
|---|---|---|
| 3181 | 8872 | C |
| 8873 | 9059 | B |
| 9059 | 9569 | C |
| 9570 | 9686 | N |

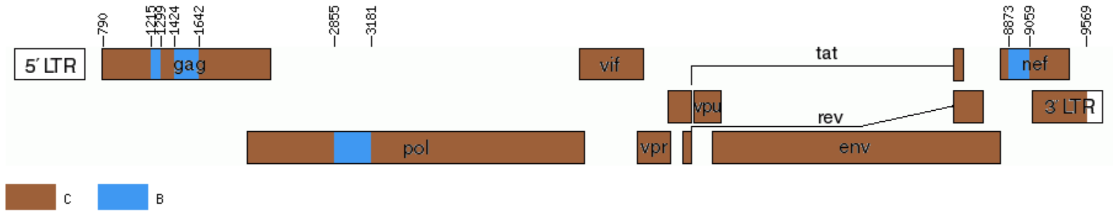


CRF57_BC

| | | |
|---|---|---|
| 1 | 789 | N |
| 790 | 1214 | C |
| 1215 | 1838 | B |
| 1839 | 2504 | C |
| 2505 | 2581 | B |
| 2582 | 4387 | C |
| 4388 | 4498 | B |
| 4499 | 5693 | C |
| 5694 | 5774 | B |
| 5775 | 6379 | C |
| 6380 | 6423 | B |
| 6424 | 9600 | C |
| 9601 | 9686 | N |

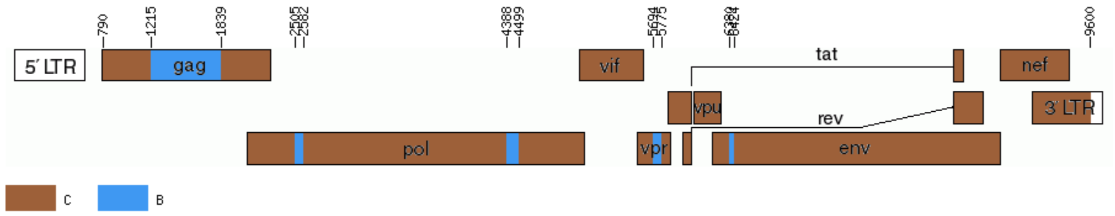


CRF61_BC

| | | |
|---|---|---|
| 1 | 789 | N |
| 790 | 1213 | C |
| 1214 | 1277 | B |
| 1278 | 2504 | C |
| 2505 | 2579 | B |

| | | |
|---|---|---|
| 2580 | 2780 | C |
| 2781 | 3181 | B |
| 3182 | 8779 | C |
| 8780 | 9078 | B |
| 9079 | 9412 | C |
| 9413 | 9689 | N |

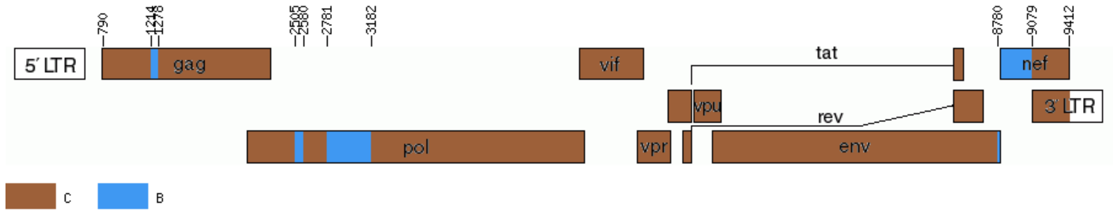


CRF62_BC

| | | |
|---|---|---|
| 1 | 789 | N |
| 790 | 2622 | C |
| 2623 | 3219 | B |
| 3220 | 5693 | C |
| 5694 | 5790 | B |
| 5791 | 6042 | C |
| 6043 | 6418 | B |
| 6419 | 9598 | C |
| 9599 | 9686 | N |

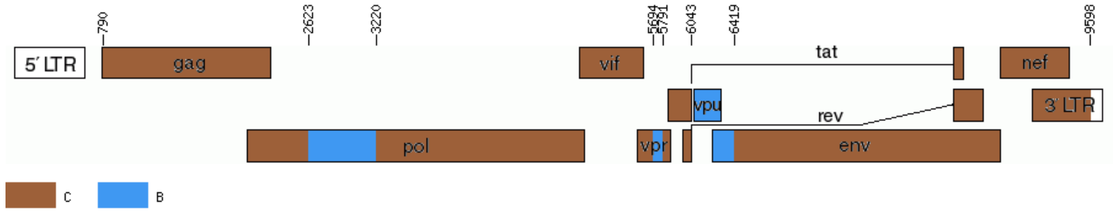


CRF64_BC

| | | |
|---|---|---|
| 1 | 789 | N |
| 790 | 1191 | C |
| 1192 | 1331 | B |
| 1332 | 1599 | C |
| 1600 | 1641 | B |
| 1642 | 1917 | C |
| 1918 | 2277 | B |
| 2278 | 2455 | C |
| 2456 | 2584 | B |
| 2585 | 4633 | C |

| | | |
|---|---|---|
| 4634 | 4919 | B |
| 4920 | 8679 | C |
| 8680 | 8752 | B |
| 8753 | 8872 | C |
| 8873 | 9009 | B |
| 9009 | 9602 | C |
| 9603 | 9686 | N |

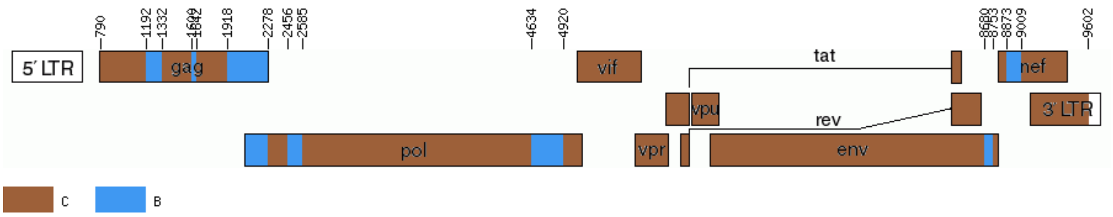

Supplement: Supplementary Table S1, Figure S1-S4 [file srep19279-s1.pdf]
